# Oxidative stress-induced endothelial cells-derived exosomes accelerate skin flap survival through Lnc NEAT1-mediated promotion of endothelial progenitor cell function

**DOI:** 10.1186/s13287-022-03013-9

**Published:** 2022-07-18

**Authors:** Linlin Guo, Yuxuan Chen, Xiaoling Feng, Di Sun, Jiaming Sun, Shan Mou, Kangcheng Zhao, Ran An

**Affiliations:** 1 Department of Plastic Surgery, Union Hospital, Tongji Medical College, Huazhong University of Science and Technology, 1277 Jiefang Avenue, Wuhan, 430022 China; 2 Wuhan Clinical Research Center for Superficial Organ Reconstruction, Wuhan, 430022 China; 3 Department of Orthopedics, Union Hospital, Tongji Medical College, Huazhong University of Science and Technology, 1277 Jiefang Avenue, Wuhan, 430022 China

**Keywords:** Oxidative stress, Exosomes, Endothelial progenitor cells, Angiogenesis, Lnc NEAT1, Skin flap

## Abstract

**Background:**

Flap transplantation is commonly used in reconstructive surgery. A prerequisite for skin flap survival is sufficient blood supply. However, such approaches remain unclear. This study aimed to explore the underlying mechanisms of exosomes derived from human umbilical vascular endothelial cells (HUVECs) exposed to oxidative stress on endothelial progenitor cells (EPCs) and their subsequent influence on the survival of skin flaps.

**Methods:**

HUVECs were treated with various concentrations of H_2_O_2_ to establish an oxidative stress model. To investigate the effects of H_2_O_2_-HUVEC-Exos and HUVEC-Exos, Cell Counting Kit-8, tube formation, invasion assays, and quantitative real-time polymerase chain reaction (qRT-PCR) were performed in EPCs. Microarray analysis was used to reveal the differentially expressed long non-coding RNAs (lncRNAs) in the H_2_O_2_-HUVEC-Exos and HUVEC-Exos. In addition, gene silencing and western blotting were employed to determine the mechanism behind lncRNA nuclear enrichment enriched transcript 1 (Lnc NEAT1) in EPCs. Further, a rat skin flap model was used to determine the role of the exosomes in skin flap survival in vivo*.*

**Results:**

HUVECs were stimulated with 100 μmol/L H_2_O_2_ for 12 h to establish an oxidative stress model. H_2_O_2_-HUVEC-Exos promoted the proliferation, tube formation, and invasion of EPCs and remarkably increased skin flap survival compared to the HUVEC-Exos and control groups. Sequencing of exosome RNAs revealed that the Lnc NEAT1 level was dramatically increased in the H_2_O_2_-HUVEC-Exos, leading to activation of the Wnt/β-catenin signaling pathway. Comparatively, knockdown of Lnc NEAT1 in HUVEC-Exos and H_2_O_2_-HUVEC-Exos significantly inhibits the angiogenic capacity of EPCs, reduced the survival area of skin flap and downregulated the expression levels of Wnt/β-catenin signaling pathway proteins, whereas Wnt agonist partly reversed the negative effect of NEAT1 downregulation on EPCs through the Wnt/β-catenin signaling pathway.

**Conclusions:**

Exosomes derived from HUVECs stimulated by oxidative stress significantly promoted the pro-angiogenic ability of EPCs through the Wnt/β-catenin signaling pathway mediated by Lnc NEAT1 and hence enhanced random flap survival in vivo. Therefore, the application of H_2_O_2_-HUVEC-Exos may serve as an alternative therapy for improving random skin flap survival.

**Supplementary Information:**

The online version contains supplementary material available at 10.1186/s13287-022-03013-9.

## Background

Skin flap techniques are extensively used in tissue reconstruction [1]. The survival of flaps after transplantation depends mainly on angiogenesis [2]. In addition, ischemia–reperfusion injury and excessive oxidative stress may initiate a cascade to damage flap tissue vascular endothelial cells and cause tissue cell apoptosis, ultimately leading to skin flap necrosis [3]. Therefore, enhancing the blood supply to the flap, relieving oxidative stress damage, and inhibiting apoptosis are crucial for improving flap viability [4, 5]. The loss of vitality of the skin flap, ranging from 9 to 65%, has a serious influence on the patient's quality of life [6]. Proangiogenic molecules (vascular endothelial growth factors, VEGF) and drugs (valproic acid and pravastatin) have been considered promising approaches to enhancing flap survival by promoting angiogenesis and inhibiting apoptosis [7–9]. However, these approaches remain controversial.

The function of exosomes in vascularization has attracted increased attention. Exosomes, a type of vesicle with a diameter of approximately 50–150 nm, are secreted from almost all cell types [10]. Exosomes are mediators that deliver functional proteins, microRNAs (miRNAs), mRNA, and non-coding RNAs (ncRNAs) to neighboring or distant cells and play a key role in regulating intercellular communication [11, 12]. Li et al. demonstrated that exosomes derived from human umbilical vascular endothelial cells (HUVECs) could induce hepatocarcinoma invasion and metastasis by promoting angiogenesis in an in vivo model [13]. Moreover, ischemic endothelial cells exhibit distinct RNA and protein profiles and angiogeneic ability compared to cells from a non-ischemic state [14]. Interestingly, exosomes derived from ischemic endothelial cells have a more robust effect on promoting axonal growth [14]. However, whether exosomes derived from human vascular endothelial cells under oxidative stress play a role in skin flap survival still needs to be further investigated.

In the last decade, long non-coding RNAs (lncRNAs) have emerged as novel regulators of biological processes. Studies have reported that lncRNAs regulate gene transcription and post-transcriptional gene expression levels by binding to DNA, RNA, or proteins and further participate in various physiological and pathological processes, such as mediating cell proliferation, migration, and apoptosis [15, 16]. LncRNA nuclear enrichment enriched transcript 1 (Lnc NEAT1) has been found to be abnormally upregulated in various human cancer types with the ability to promote tumor growth [17]. It has been reported that Lnc NEAT1 mediated by exosomes can facilitate endometrial cancer progression [18]. Recent evidence indicates that Lnc NEAT1 regulates stem cell function [19, 20]. Nevertheless, the roles of Lnc NEAT1 in EPC function and skin flap survival remain unknown.

Endothelial progenitor cells (EPCs), which are a type of bone marrow-derived circulating progenitor cells for the endothelial lineage, have garnered considerable interest in their role in neovascularization and vascular repair [21, 22]. EPCs have been reported to be able to migrate to the hypoxic/ischemic sites where they secrete a cascade of angiogenic factors, transdifferentiate into mature endothelial cells, and participate in angiogenesis to form new blood vessels [23–25]. Furthermore, EPCs have the ability to prevent endothelial cells from apoptosis and preserve their angiogenic potential under oxidative stress conditions [22]. A previous study demonstrated that transplantation of EPCs could facilitate thrombus resolution by altering the vein microenvironment [26]. However, the number of EPCs in peripheral blood is limited. Therefore, it is critically important to improve the recruitment of EPCs to disease sites and enhance the angiogenic potential of EPCs.

In the present study, we aimed to observe the effect of exosomes derived from HUVECs stimulated by oxidative stress and under normal conditions on EPC functions and the changes in Lnc RNA profiles. Herein, we identified that exosomal Lnc NEAT1 can be transferred to EPCs, thereby promoting EPC angiogenesis via the Wnt/β-catenin signaling pathway. We further demonstrated that the transplantation of exosomal cargo containing Lnc NEAT1 facilitated the survival of skin flaps in a rat modified McFarlane flap model. Thus, our results suggest that HUVEC exosomal Lnc NEAT1 acts as a promising therapeutic approach for skin flap tissue repair.

## Methods

### Cell culture

Human umbilical vein endothelial cells (HUVECs) were purchased from Cell Bank of Chinese Academy of Sciences (Shanghai, China). HUVECs were grown in RPMI 1640 medium (HyClone, Logan, UT, USA) supplemented with 10% fetal bovine serum (Evergreen, Hangzhou, Zhejiang, China) and 1% penicillin–streptomycin solution (Biosharp, Hefei, Anhui, China) and maintained at 37 °C in a 5% CO_2_ humidified atmosphere.

Umbilical cord blood was collected from donors with informed consent, which was approved by the Ethics Committee of Huazhong University of Science and Technology. The mononuclear cells were fractionated from the umbilical cord blood by gradient centrifugation with human peripheral blood lymphocyte separation solution (TBD, Tianjin, China) according to the manufacturer’s instructions. The isolated mononuclear cells were seeded onto cell culture dishes coated with 0.01% human fibronectin (Solarbio, Beijing, China) at a density of 5 × 10^6^ cells/cm^2^ in EGM-2 BulletKit (Lonza, Basel, Switzerland), and maintained at 37 °C in a 5% CO_2_ humidified atmosphere. EPCs from passages 1 to 3 were used in the subsequent studies.

### Cell counting kit-8 (CCK8)

HUVECs and EPCs were seeded in 96-well plates (10^3^ cells/well). HUVECs were treated with different concentrations of H_2_O_2_ (0, 50, 100, 200, 400 μmol/L) for 8,12 and 24 h, respectively. EPCs were co-cultured with different concentrations of exosomes (HUVEC-Exos and H_2_O_2_-HUVEC-Exos: 25, 50,100 μgm/L) for 24 h. After washing three times with PBS, 10 μl CCK-8 reagent (Beyotime Biotechnology, Shanghai, China) was added to each well and incubated at 37 °C for 1 h. The absorbance was measured at 450 nm using a microplate reader (BioTek, VT, USA).

### Superoxide dismutase (SOD) activity assay

HUVECs were seeded in 10-cm dishes (10^6^ cells/dish) and then treated with different concentrations of H_2_O_2_ (0, 50, 100, 200, and 400 μmol/L) for 8, 12, and 24 h, respectively. The total Superoxide Dismutase Assay Kit with WST-1(Jiancheng Biotech Ltd., Nanjing, China) was used to detect SOD activity according to the manufacturer's instructions.

### Flow cytometry analysis

HUVECs were treated with various concentrations of H_2_O_2_ (0, 50, 100, 200, and 400 μmol/L). After treatment, cells were harvested and washed with PBS. Apoptosis was detected using an FITC Annexin V Apoptosis Detection kit (Catalog no: 556547, BD Biosciences, San Jose, CA, USA). The upper left quadrant represents a mechanically injured cell, and the upper right quadrant represents a late apoptotic cell. The lower left quadrant is a normal cell, and the lower right quadrant is an early apoptotic cell. In this experiment, the proportion of the upper right quadrant and lower right quadrant was used as the percentage of apoptotic cells. The data were analyzed using the FlowJo software (version 10.7.1, Stanford University, USA).

To identify EPCs, 1 × 10^6^ EPCs were used for each antibody in a separate tube and incubated with 20 μl of antibodies for 30 min on ice. The cells were washed three times with PBS to remove unbound antibodies. The cell suspension was aspirated and analyzed by flow cytometry (BD LSRFortessa^™^ X-20, San Jose, CA, USA). Flow cytometry analysis was performed in triplicate for each sample. The following antibodies were used: fluorescein isothiocyanate (FITC)-conjugated CD34 (Catalog no: 555822), phycoerythrin (PE) conjugated-CD133 (Catalog no: 566595), allophycocyanin (APC) conjugated-CD309 (Catalog no: 560495), PE conjugated-CD14 (Catalog no: 345785), APC conjugated-CD45 (Catalog no: 557659), all antibodies were purchased from BD Biosciences, San Jose, CA, USA).

### Dil-Ac-LDL/ FITC-UEA-1 staining

The adherent cells were incubated in a medium containing Dil-Ac-LDL (2.4 μg/ml; MKBio, Shanghai, China) at 37 °C for 1 h, washed twice, and fixed in 4% paraformaldehyde for 10 min. The cells were then incubated in FITC-UEA-1 lectin (10 μg/ml; MKBio, Shanghai, China) for 1 h. 4', 6-diamidino-2-phenylindole (DAPI, Beyotime Biotechnology, Shanghai, China) was used to observe the cell nuclei. Images were acquired using a confocal microscope (Nikon, Tokyo, Japan). Double-positive staining for DiI-Ac-LDL and FITC-UEA-1 lectin was considered an EPC.

### Isolation of exosomes derived from HUVECs

HUVECs were treated with 100 μmol/L H_2_O_2_ serum-free RPMI 1640 medium (HyClone, USA) and serum-free RPMI 1640 medium for 12 h. Then, the two groups of HUVECs were cultured in serum-free medium for 72 h. Additionally, after transfecting HUVECs and H_2_O_2_-HUVECs (treated with 100 μmol/L H_2_O_2_ for 12 h) with NEAT1 siRNA lentivirus, the two groups of cells were also cultured in serum-free medium for 72 h before extracting the supernatant. Thereafter, exosomes were extracted from the four groups of HUVECs and the collected medium was centrifuged at 300** × **g for 10 min to remove the cell pellet. and then subjected to centrifugation at 2000** × **g for 10 min and at 10,000** × **g for 30 min, followed by a 0.22 μm sterilized filter (MilliporeExpress^®^ PES membrane, Millex, Bedford, MA, USA) to remove larger diameter vesicles. The supernatants were centrifuged at 110,000** × **g for 70 min, and the extracted exosomes were re-suspended in pre-cooled PBS. To evaluate the exosomal concentration, exosomes were lysed in radio-immunoprecipitation assay (RIPA) lysis buffer, and then a bicinchoninic acid (BCA) protein assay kit (Servicebio, Wuhan, Hubei, China) was applied to detect protein concentration in exosomes according to the manufacturer's instructions.

### Internalization of exosomes

To visualize the internalization of exosomes in EPCs, exosomes were stained with DiI dye (Solarbio, Beijing, China). After the DiI-exosomes were co-cultured with EPCs for 4 h, the EPCs were stained with Calcein AM (Beyotime Biotechnology, Shanghai, China) and DAPI (Beyotime Biotechnology, Shanghai, China) to visualize the cytoplasm and nucleus, respectively. The uptake was imaged using a confocal microscope (Nikon, Tokyo, Japan).

### Nanoparticle tracking analysis (NTA)

Nanoparticle tracking analysis using a ZetaView (Particle Metri, Bavaria, Germany) was used to analyze the particle size distribution and concentration of exosomes. When the exosomes were irradiated by a blue laser (488 nm), the movement of exosomes under Brownian motion was recorded in videos. Three videos of 60 s were taken, and particles sizes were analyzed by NTA software analyzed using the in-build ZetaView Software (version 8.02.31, Particle Metri, Bavaria, Germany).

### Transmission electron microscope (TEM)

Exosomes diluted in PBS were dropped on the red wax, and a polymethyl vinyl acetate/carbon-coated copper mesh was placed in the droplet and allowed to stand at room temperature for 20 min. The filter paper absorbed excess liquid, and the sample was fixed with 2% paraformaldehyde (PFA) for 2 min. The copper mesh was washed three times with double distilled water and counterstained with 2% phosphotungstic acid (PTA) for 1 min. The filter paper removed the excess liquid from the copper mesh and dried overnight at room temperature. Imaging was performed using an electron microscope (Tecnai G2 20, Thermo Fisher Scientific, Cleveland, OH, USA).

#### Transwell assay

A transwell chamber was used (24-well plate, JETBIOFIL, Guangzhou, Guangdong, China) for the invasion assay. 200 μl of serum-free medium containing 5** × **10^3^ EPCs was added to the upper chamber, and 600 μl of serum-free medium with different concentrations of exosomes (control group: 0 μg/ml, HUVEC-Exos group or H_2_O_2_-HUVEC-Exos group: 25, 50, and 100 μg/ml; si-NEAT1-HUVEC-Exos: 100 μg/ml, si-NEAT1-H_2_O_2_-HUVEC-Exos group: 100 μg/ml, si-NEAT1-HUVEC-Exos (100 μg/ml) + HLY78 (20 μg/ml, MedChemExpress, NJ, USA), si-NEAT1-H_2_O_2_-HUVEC-Exos (100 μg/ml) + HLY78 (20 μg/ml)) was added to the lower chamber. After incubation for 24 h, non-migrating cells above the filter were wiped off with a cotton swab. The filters were fixed with 4% paraformaldehyde and stained with DAPI (Beyotime Biotechnology, Shanghai, China). Invaded cells were quantified in three representative microscopic fields at 200** × **magnification with three fields per chamber.

#### Tube formation assay

EPCs (1** × **10^4^) were added to a 96-well culture plate coated with Matrigel (50 μl/well, BD Biosciences, San Jose, CA, USA) in EPC basic medium with different concentrations of exosomes (HUVEC-Exos group or H_2_O_2_-HUVEC-Exos group: 25, 50, and 100 μg/ml; si-NEAT1-HUVEC-Exos: 100 μg/ml, si-NEAT1-H_2_O_2_-HUVEC-Exos group: 100 μg/ml, si-NEAT1-HUVEC-Exos (100 μg/ml) + HLY78 (20 μg/ml, MedChemExpress, NJ, USA), si-NEAT1-H_2_O_2_-HUVEC-Exos (100 μg/ml) + HLY78 (20 μg/ml)) or PBS (control group). After 8 h of incubation, tube formation was observed under a confocal microscope (Nikon, Tokyo, Japan) at 40** × **magnification with at least four fields. The number of nodes, branches, and total branch length was measured using ImageJ (National Institutes of Health, USA).

#### Western blot analysis

Total protein extracts were quantified using a BCA assay kit (Thermo Fisher Scientific, Cleveland, OH, USA), separated by 10% sodium dodecyl sulfate polyacrylamide gel electrophoresis (SDS-PAGE), and then transferred to nitrocellulose membranes. The immunoblots were blocked with 5% fat-free dry milk at room temperature for 1 h and incubated with primary antibodies overnight at 4 °C, followed by incubation with horseradish peroxidase (HRP)-conjugated secondary antibodies for 1 h at room temperature. Protein bands were visualized by enhanced chemiluminescence. Band intensities were analyzed by densitometry using Image-Pro Plus (Media Cybernetics, USA). β-Actin was used as an internal control. The following primary antibodies were used: anti-human CD63 antibody (1:1000, AF5117, Affinity, Melbourne, Australia), anti-human CD81 antibody (1:1000, DF2306, Affinity, Melbourne, Australia), anti-human calnexin antibody (1:1000, AF5362, Affinity, Melbourne, Australia), anti-human β-actin antibody (1:1000, 66,009-Ig, Abcam, Cambridge, UK), anti-human β-catenin antibody (1:1000, ab32572, Abcam, Cambridge, UK), anti-human c-myc antibody (1:1000, Af0358, Affinity, Melbourne, Australia), and anti-human cyclin D1 antibody (1:1000, ab16663, Abcam, Cambridge, UK).

#### Quantitative real-time polymerase chain reaction (qRT-PCR)

Total RNA from cell samples was extracted using TRIzol (Invitrogen, Carlsbad, CA, USA) and reverse transcribed into cDNA using a first-strand cDNA synthesis kit (Thermo Fisher Scientific, Cleveland, OH, USA) according to the manufacturer's protocol. All reactions were performed in triplicate. qRT-PCR was performed under the following thermal conditions: 95 °C for 10 min, followed by 40 cycles of 95 °C for 15 s, 60 °C for 60 s, and 72 °C for 60 s. Glyceraldehyde-3-phosphate dehydrogenase (GAPDH) was used as a normalization control. All results were calculated using the 2-ΔΔCt method and normalized to GAPDH. The primers used were as follows:

hsa- vascular endothelial growth factor A (VEGFA): forward, ATCCAATCGAGACCCTGGTG, and reverse, ATCTCTCCTATGTGCTGGCC;

hsa- platelet derived growth factor C (PDGFC): forward, GAACTGTGCCTGTTGTCTCC, and reverse, ACACACAGTCACACTCCTCA;

has- prime time entertainment network (PTEN): forward, CACGACGGGAAGACAAGTTC, and reverse, GGTTTCCTCTGGTCCTGGTA;

hsa-NEAT1: forward, GCCTTGTAGATGGAGCTTGC, and reverse, GCACAACACAATGACACCCT.

#### Establishment of skin flap model

All procedures were approved by the guidelines of the Animal Research Committee of the Huazhong University of Science and Technology. All experiments involving animals were purchased from Hubei Bainte Biological Technology Co., Ltd. (China). Twenty-five male Sprague–Dawley rats (8 weeks old, weighing 400–450 g) were used. All rats were randomly divided into five groups: control group (*n* = 5), HUVEC-Exos group (*n* = 5), H_2_O_2_-HUVEC-Exos group (*n* = 5), si-NEAT1-H_2_O_2_-HUVEC-Exos group (*n* = 5), and si-NEAT1-HUVEC-Exos group (*n* = 5). After anesthesia with pentobarbital sodium (6 mg/100 g intraperitoneally), the dorsal midline was used as the longitudinal axis, and the pedicle was located on the bilateral iliac crest articulation to design a rectangular flap area (3** × **12 cm) in each rat. Each flap was divided into three equally sized zones: proximal (area I), intermediate (area II), and distal (area III) zones. The skin and subcutaneous tissue were separated from the underlying fascia as previously reported [9]. All blood vessels were completely incised, and each flap was immediately returned and sutured in situ with 4–0 non-absorbable sutures. After the operation, 500 µg of different types of DiR-labeled exosomes (HUVEC-Exos, H_2_O_2_-HUVEC-Exos, si-NEAT1-HUVEC-Exos, si-NEAT1-H_2_O_2_-HUVEC-Exos) in 200 µl PBS or 200 µl PBS alone (control) were injected into the tail vein immediately. Fourteen days after the operation, the rats were killed for histological examination.

#### Flap survival evaluation

After surgery, the texture, tissue elasticity, and color of the flaps were monitored continuously for 14 days. The following criteria were used to identify necrosis: rigid texture, poor elasticity, black flap, and no bleeding when cutting the flap. The rats were killed on day 14. The necrotic and living flap areas were measured. The percentage of survival area was calculated as follows: survival area/total area of flap** × **100%.

#### In vivo fluorescence imaging

For in vivo fluorescence imaging, the rates were anesthetized and the distribution of exosomes was tracked using a Bruker In-Vivo Xtreme (Bruker, Karlsruhe, Baden-Württemberg, Germany) at 30 min, 4 h, 3 d, and 7 d after exosome injection. 750 nm excitation filter and 790 nm emission filter were used to detect the fluorescence signal, and the exposure time was set to 10 s. Fluorescence images were analyzed using Bruker MI SE 721 software (Bruker, Karlsruhe, Baden-Württemberg, Germany).

#### Histological, immunohistochemistry analysis and immunofluorescence staining

On postoperative day 14, the rats were killed. Tissue samples (1** × **1 cm) were collected from the center of area II from the skin flap, fixed in 4% paraformaldehyde, dehydrated, embedded in paraffin, and sectioned into 4-um-thick slices. The sections were stained with hematoxylin and eosin (H&E) and photographed using an optical microscope. The sections were incubated with anti-human CD31 antibodies (1:100, bs-0468R, Bioss, Beijing, China) and anti-human α-SMA antibody (1:100, BM0002, Boster Biological Technology, Pleasanton, CA, USA) at 4 °C overnight, developed with 3, 3’-diaminobenzidine tetrahydrochloride (DAB) and counterstained with hematoxylin. Representative pictures were observed at 40** × **magnification. magnification using a fluorescence microscope (Nikon, Tokyo, Japan). Three sections and three fields from each section of each sample were randomly selected and examined. CD31 and α-SMA staining were used to evaluate the formation of newly formed blood vessels and mature vessels, respectively. The number of CD31 positive vessels and α-SMA-positive vessels was determined using ImageJ (National Institutes of Health, USA).

For immunofluorescence staining, the sections were incubated with primary antibodies against CD34 (1:2000, ab81289, Abcam, Cambridge, UK) and CD133 (1:1000, 66,666–1-IG, Proteintech, Wuhan, China) overnight at 4 °C, followed by incubation with secondary antibodies (anti-CD34: 1:2000, ab81289, Abcam, anti:CD133: 1:1000, ab6789, Abcam, Cambridge, UK) at room temperature for 1 h in the dark. Images were acquired using a confocal microscope (Nikon, Tokyo, Japan). The number of CD34/CD133 double-positive cells was analyzed using ImageJ software (National Institutes of Health, USA).

#### RNA sequencing assay

Total RNA was extracted from the samples using TRIzol reagent (Invitrogen, Carlsbad, CA, USA). The total RNA of the samples was quantified using a NanoDrop ND-2000 (Thermo Scientific, Cleveland, OH, USA), and RNA integrity was checked using an Agilent Bioanalyzer 2100 (Agilent Technologies, Santa Clara, CA, USA). The QIAGEN RNeasy Kit (Qiagen, Hilden, Germany) was used to remove ribosomal RNA from total RNA to enrich non-coding RNA. After amplification by PCR and quantification using the Agilent Bioanalyzer 2100 system (Agilent Technologies, Santa Clara, CA, USA), the cDNA library of lncRNA was sequenced on an Agilent Scanner G5761A (version 14.8, Agilent Technologies, Santa Clara, CA, USA). The *P* value of the T test was required to screen the differentially expressed lncRNAs, and the selection criterion was a *P* value ≤ 0.05. Volcano plots and heat maps were generated using R software (version 4.1.2, USA) using R packages "ggplot2" and "Pheatmap".

#### Transfection assay

HUVECs and H_2_O_2_-HUVECs were seeded onto 6-well plates and cultured overnight. When the fusion rate of logarithmic growth cells reached 70–90%, HUVECs and H_2_O_2_-HUVECs were transfected with NEAT1 siRNA lentivirus, synthesized by Wuhan Biofavor Biothech Service Co., using Lipofectamine 3000 Transfection Reagent (Invitrogen, Carlsbad, CA, USA). The sequences were as follows: si-NEAT1: forward, GCCTTGTAGATGGAGCTTGC, and reverse, GCACAACACAATGACACCCT. PCR was used to verify the inhibition efficiency of si-NEAT1 and used these cells for subsequent functional experiments.

#### Statistical analysis

All experiments were independently repeated at least thrice. The results are expressed as mean ± SD. One-way analysis of variance (ANOVA) and unpaired t-test were used to assess the differences between groups using GraphPad Prism software (version 7.0, GraphPad Software, USA). Statistically significant difference was concluded at *P < 0.05, **P < 0.01, ***P < 0.001, ****P < 0.0001, ^#^*P* < 0.05, ^##^*P* < 0.01 and ^###^*P* < 0.001; ns. represents no statistically significant difference.

## Results

### Establishment of an oxidative stress model of HUVECs stimulated with H_2_O_2_

**Fig. 1:**
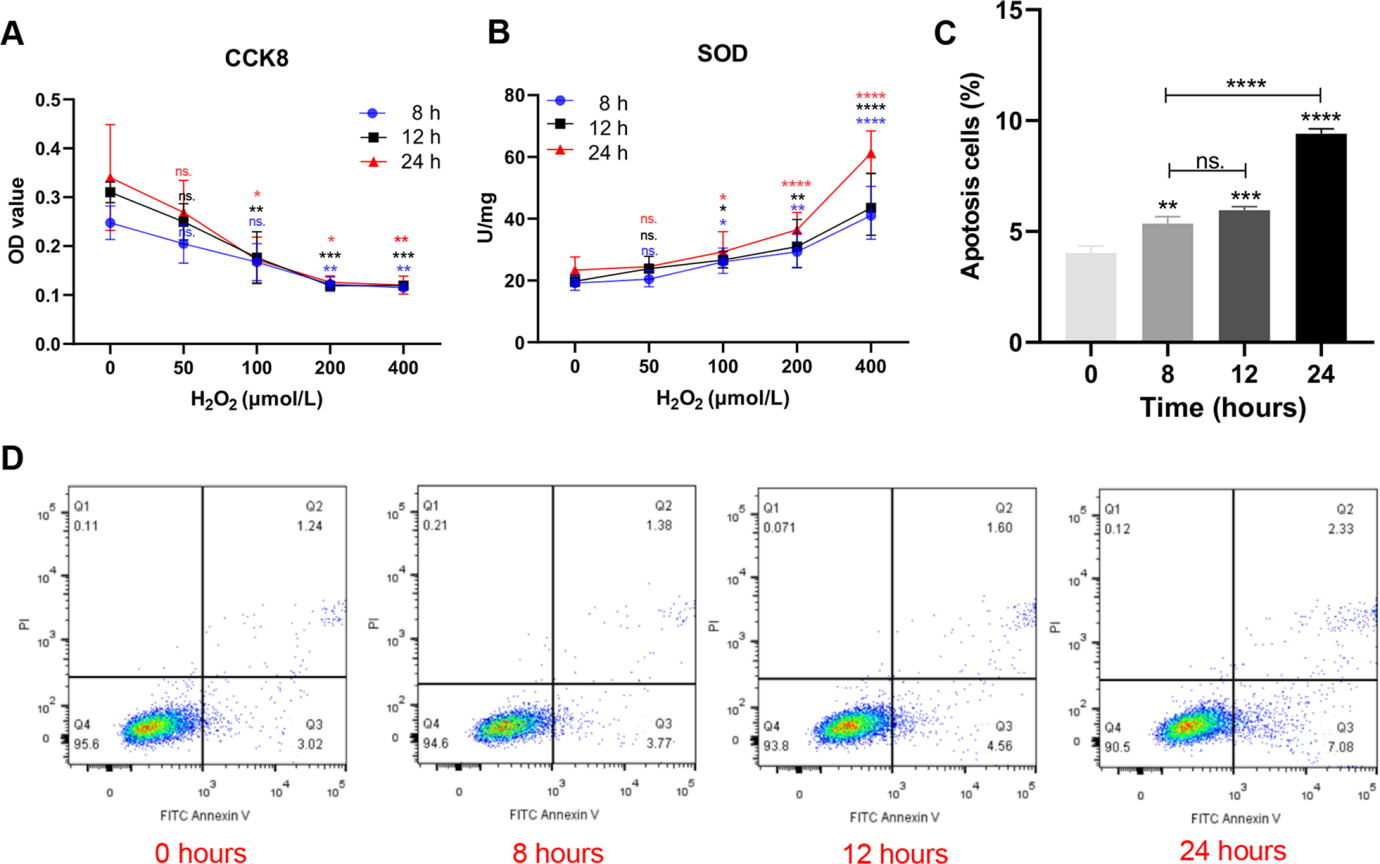
The effect of different concentrations of H_2_O_2_ on HUVECs activity and apoptosis. **A** The proliferation of HUVECs after treated with graded concentrations (0, 100, 200, and 400 μmol/L) of H_2_O_2_ at 8, 12, and 24 h measured by CCK8 assay. ns. *P* > 0.05, * *P* < 0.05, ** *P* < 0.01, *** *P* < 0.001 vs. 0 μmol/L. **B** The level of SOD viability of HUVCEs cultured with graded concentrations (0, 100, 200, and 400 μmol/L) of H_2_O_2_ at 8, 12 and 24 h. ns. *P* > 0.05, * *P* < 0.05, ** *P* < 0.01, **** *P* < 0.0001 vs. 0 umol/L. **C**, **D** The apoptosis of HUVECs after treated with 100 μmol/L H_2_O_2_ at different time points determined with Annexin V/PI staining by flow cytometry analysis. ns. *P* > 0.05, ** *P* < 0.01, *** *P* < 0.001, **** *P* < 0.0001 vs. 0 h

To determine the appropriate H_2_O_2_ concentration and conditions for constructing an oxidative stress model, HUVECs were treated with graded concentrations of H_2_O_2_ (0, 50, 100, 200, and 400 μmol/L) for three different time points (8, 12, and 24 h). CCK8 results showed that cell proliferation decreased in a dose-dependent manner (Fig. 1A). In addition, the SOD activity increased with increasing time and concentration of H_2_O_2_ (Fig. 1B). When the concentration of H_2_O_2_ exceeded 100 μmol/L to 200 and 400 μmol/L, the cell viability decreased sharply by approximately 50% and SOD vitality increased significantly (*P* < 0.05). Therefore, high concentrations of H_2_O_2_ (≥ 200 μmol/L) are not suitable for building oxidative stress models based on the lower cell viability and oxidative stress degree. Conversely, low concentrations of H_2_O_2_ (50 μmol/L) had no significant effect on the SOD viability and viability of HUVECs compared to that under the condition of 0 μmol/L H_2_O_2_ (*P* < 0.05). Interestingly, we found that the time change had little effect on cell proliferation and SOD activity at a concentration of 100 μmol/L. Based on the above, H_2_O_2_ at a concentration of 100 μmol/L was selected for the following experiment. Annexin V-FITC/PI flow cytometry was performed to detect the apoptosis rate of HUVECs stimulated with 100 μmol/L H_2_O_2_ at different time points (0, 8, 12, and 24 h). As shown in Fig. 1C, cell apoptosis was remarkably increased at 8, 12, and 24 h compared to that at 0 h. However, no significant difference in the apoptosis rate between 8 and 12 h time points was observed (Fig. 1C). However, when the incubation time was prolonged to 24 h, the apoptosis rate increased significantly compared to that at other time points (*P* < 0.0001). To facilitate further experiments, we selected 100 μmol/L H_2_O_2_ to stimulate HUVECs for 12 h to establish an oxidative stress model. Compared with HUVECs cultured with serum (serum -HUVECs), HUVECs treated with serum-free medium containing 100 umol/L H_2_O_2_ (H_2_O_2_-serum-free-HUVECs) for 12 h became larger and rounder, but the morphology of HUVECs without oxidative stress treatment (serum-free-HUVECs) did not change significantly (Additional file 1: Fig. S1A). The proliferation activity and number of H_2_O_2_-serum-free-HUVECs and serum-free-HUVECs remained above 50% of those of serum- HUVECs during the whole process (Additional file 1: Fig. S1B–C).

### Isolation and characterization of exosomes

**Fig. 2:**
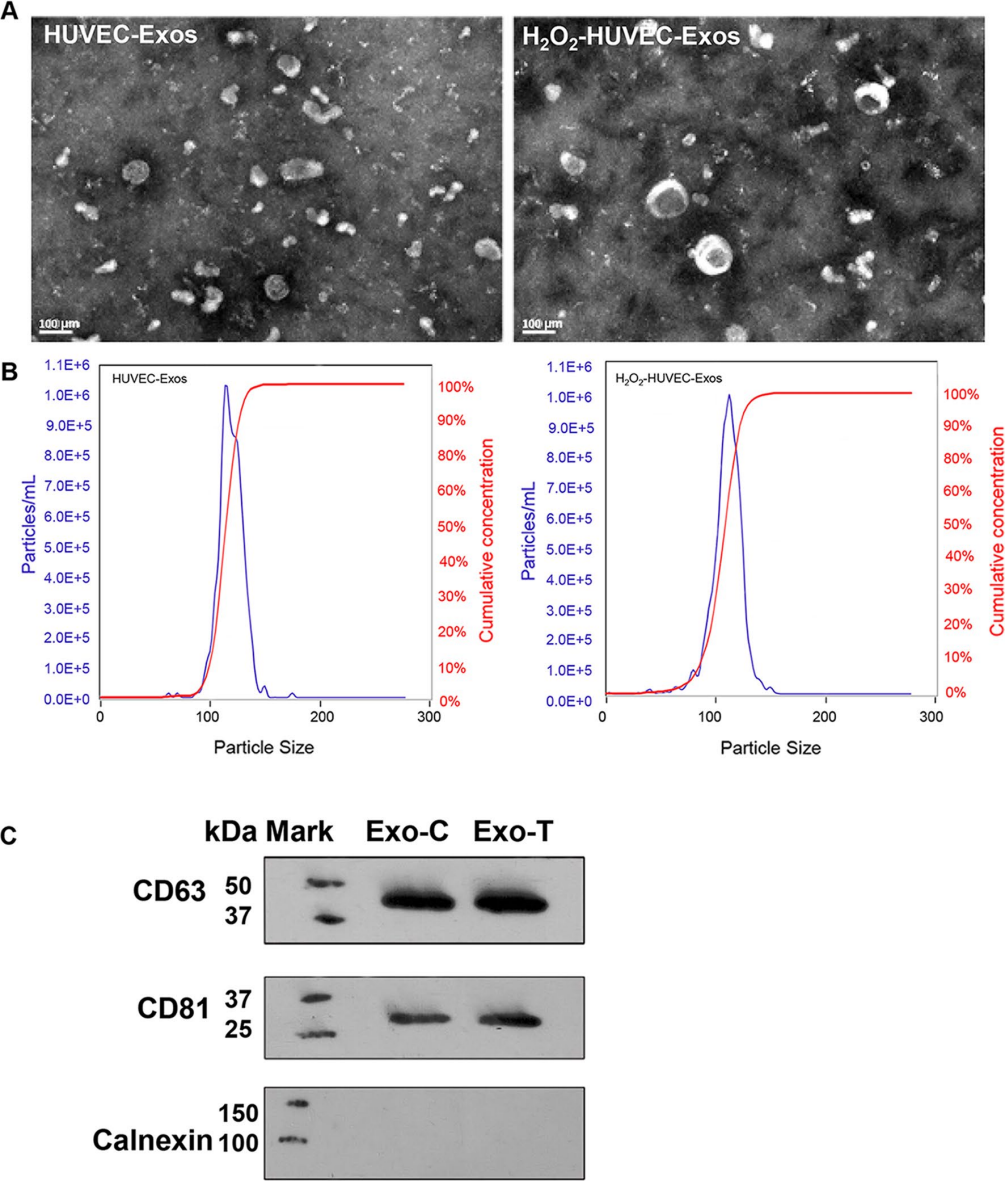
Isolation and characterization of exosomes. **A** Transmission electron micrograph of HUVEC-Exos and H_2_O_2_-HUVEC-Exos. **B** The particle size, particle concentration of HUVEC-Exos and H_2_O_2_-HUVEC-Exos were analyzed by nanoparticle tracking analysis. **C** Western blot analysis of CD63, CD81 and calnexin proteins in exosomes

Exosomes were derived from HUVECs with or without stimulation with 100 μmol/L H_2_O_2_ for 12 h. TEM revealed that the isolated exosomes from the supernatants of HUVECs with or without H_2_O_2_ treatment were spheroidal microvesicles (Fig. 2A). NTA result showed that the size of HUVEC-Exos and H_2_O_2_-HUVECs-Exos mainly ranged from 50 to 150 nm, with a mean size of 114.3 and 109.9 nm, and the two kinds of exosomes possessed similar cumulative concentration curves (Fig. 2B). Therefore, the particle size and concentration did not show significant differences between H_2_O_2_-HUVECs-Exos and HUVEC-Exos. We further investigated the relation of the number of exosomes and amount of protein in these two kinds of exosomes. Compared with HUVEC-Exos, H_2_O_2_-HUVEC-Exos possessed fewer numbers at the same protein amount (Additional file 2: Fig. S2 and Additional file 3: Table S3, *P* < 0.05). Our experiments demonstrated that H_2_O_2_ stimulation increased the protein amount in exosomes produced by HUVECs remarkably. Western blotting further verified that both the HUVEC-Exos and H_2_O_2_-HUVEC-Exos were positive for specific exosomal proteins (CD63 and CD81), but negative for calnexin (Fig. 2C).

### Characterization of EPCs

**Fig. 3:**
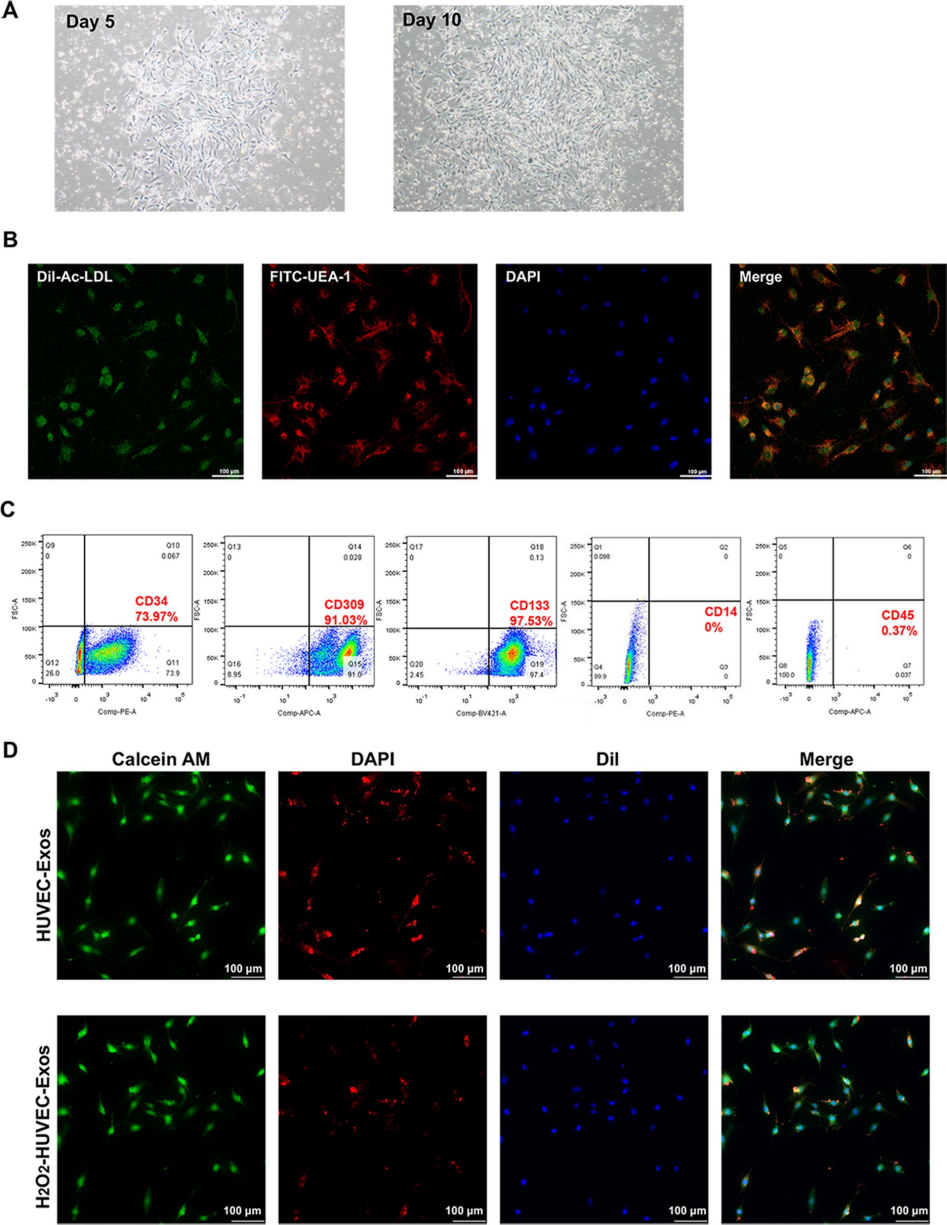
Characterization of EPCs and internalization of HUVEC-derived exosomes by EPCs. **A** Morphology of primary EPCs on the day 5 and 10 of culture. **B** Fluorescence microscopy evaluation of DiI-Ac-LDL uptake and membrane binding of FITC-UEA-1 by EPCs. DiI-Ac-LDL in red, FITC-UEA-1 in green, DAPI in blue. **C** Flow cytometry analysis of surface markers of EPCs. Positive expression of CD34, CD309, and CD133, while negative for CD14 and CD45. **D** Exosomes derived from HUVECs could be taken up by EPCs. Calcein AM (green) and DAPI (blue) were used to stain the cytoplasm and nucleus of EPC, respectively. Exosomes were labeled with DiI (red)

Adherent cells appeared as colonies and exhibited a typical spindle shape on day 5. After approximately 10 days of culture, these cells showed a typical cobblestone-like appearance (Fig. 3A). To identify the isolated EPCs by density gradient centrifugation, flow cytometry and confocal microscopy were used. As shown in Fig. 3B, confocal microscopy revealed that the isolated cells had the ability to take up DiI-Ac-LDL and were bound by FITC-UEA-1 on their membrane. The results of flow cytometry showed that the cells highly expressed CD34 (73.97%) CD309 (VEGF-R2, 91.03%) and CD133 (97.53%), while negative for CD14 (0%) and CD45 (0.37%) (Fig. 3C). These findings confirmed the successful isolation of EPCs for subsequent studies.

### Internalization of HUVEC-derived exosomes by EPCs

To determine whether the HUVEC-derived exosomes could be internalized into EPCs, DiI-labeled exosomes were incubated with EPCs for 4 h. The uptake of exosomes in EPCs was observed using confocal microscopy. As shown in Fig. 3D, the DiI-labeled exosomes were mainly localized in the cytoplasm of EPCs, and EPCs were DiI-positive in both groups.

### H_2_O_2_-HUVEC-Exos enhanced the proangiogenic abilities of EPCs

**Fig. 4:**
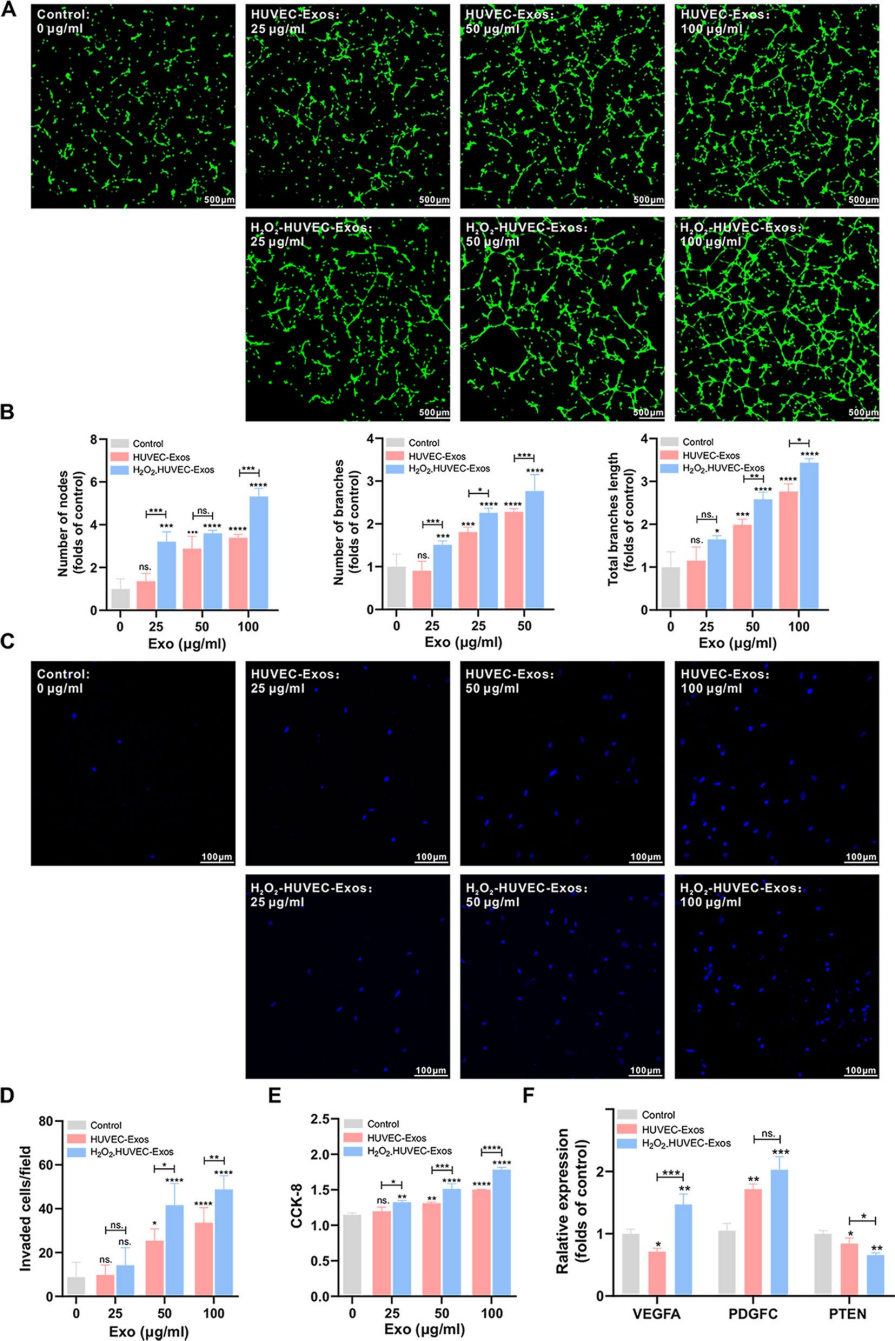
H_2_O_2_-HUVEC-Exos enhanced the pro-angiogenic abilities of EPCs in a dose-dependent manner. **A** Representative images of capillary-like tube formation of EPCs incubated with graded concentration (0–100 μmol/L) of HUVEC-Exos and H_2_O_2_-HUVEC-Exos or PBS. EPCs were labeled with Calcein-AM (green). **B** Quantitative analysis of the number of nodes, the number of branches and the total length of the branches of (A). **C** Representative images of invaded EPCs stained by DAPI after cultured with graded concentration (0–100 μmol/L) of HUVEC-Exos and H_2_O_2_-HUVEC-Exos or PBS. **D** Quantitative analysis of (C). **E.** The proliferation of EPCs treated with graded concentration (0–100 μmol/L) of HUVEC-Exos and H_2_O_2_-HUVEC-Exos or PBS detected by CCK8. **F** The VEGF, PDGFC, and PTEN mRNA expression of EPCs after treated with 100 μmol/L HUVEC-Exos and H_2_O_2_-HUVEC-Exos. ns. *P* > 0.05, * *P* < 0.05, ** *P* < 0.01, *** *P* < 0.001, **** *P* < 0.0001 vs. control group

To detect the pro-angiogenic effect of exosomes derived from HUVECs on EPCs, a tube formation assay was performed. As shown in Fig. 4A, B, compared to the control and HUVEC-Exos groups, EPCs co-cultured with H_2_O_2_-HUVEC-Exos generated higher numbers of nodes, number of branches, and total length of branches, and the effect increased in a dose-dependent manner, with the highest concentration (100 μg/ml) showing the strongest effect. Subsequently, a transwell assay was performed to evaluate the migration ability of EPCs. No statistical difference in the invasion of EPCs was found after treatment with low concentrations of exosomes (25 μg/ml, both HUVEC-Exos and H_2_O_2_-HUVEC-Exos) treatments compared with the control group (Fig. 4C, D, *P* > 0.05). When the concentration of exosomes reached 50 and 100 μg/ml, H_2_O_2_-HUVEC-Exos remarkably enhanced the invasion of EPCs by 1.64 and 1.45-fold, respectively, compared to the HUVEC-Exos group (*P* < 0.05). As shown in Fig. 4E, the ability of exosomes (both HUVEC-Exos and H_2_O_2_-HUVEC-Exos) to promote cell proliferation was concentration-dependent, with the highest proliferation observed in the presence of 100 μg/ml H_2_O_2_-HUVEC-Exos (*P* < 0.05). PCR analysis was conducted to detect the mRNA expression levels of pro-angiogenic genes (VEGFA and PDGFC) and anti-angiogenic genes (PTEN). The expression of VEGFA in the H_2_O_2_-HUVEC-Exos group was significantly upregulated, which was accompanied by a significant downregulation of PTEN compared to the other groups (Fig. 4F,* P* < 0.05). With regard to PDGFC expression, no significant difference was observed between HUVEC-Exos and H_2_O_2_-HUVEC-Exos groups (Fig. 4F,* P* > 0.05), but both enhanced the expression of PDGFC significantly compared to the control (Fig. 4F,* P* < 0.01).

### Lnc NEAT1 was significantly upregulated in H_2_O_2_-HUVEC-Exos

**Fig. 5:**
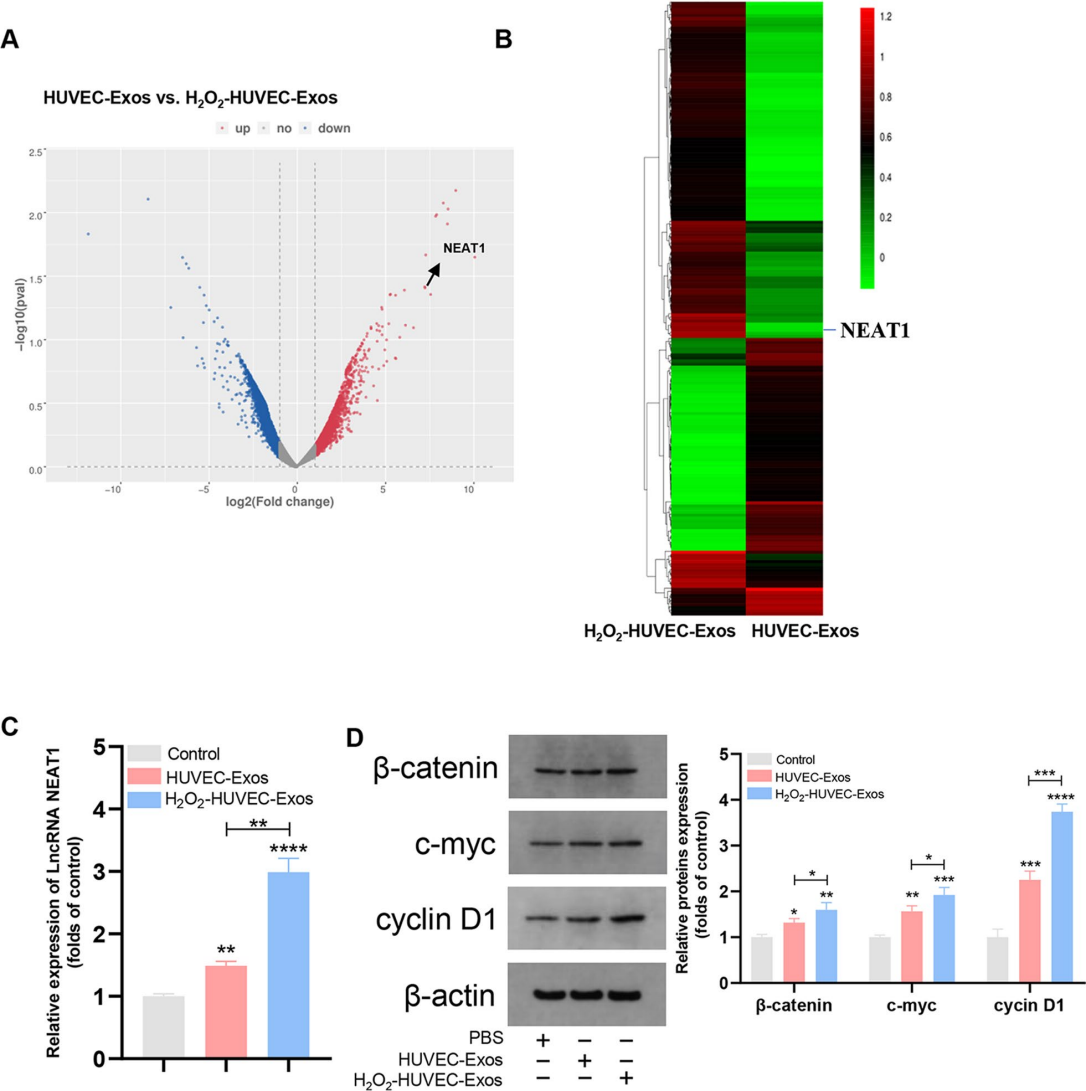
The differential expression of Lnc RNAs in exosomes and proteins in the Wnt/β-catenin pathway. **A** The volcano plot represented the differential expression of lncRNAs. The blue dots represent the downregulated genes, while the red dots represent upregulated genes. **B** Heat map of differential expression of Lnc RNAs in H_2_O_2_-HUVEC-Exos and HUVEC-Exos. Red corresponds to upregulated LncRNAs, and green corresponds to downregulated LncRNAs. **C** Quantification of Lnc NEAT1 level in EPCs after cultured with PBS, HUVEC-Exos, or H_2_O_2_-HUVEC-Exos. **D** The β-catenin, c-myc and cyclin D1 protein levels in EPCs in control, HUVEC-Exos or H_2_O_2_-HUVEC-Exos group. * *P* < 0.05, ** *P* < 0.01, *** *P* < 0.001, **** *P* < 0.0001 vs. control group

Differential expression of lncRNAs between different groups was screened by microarray analysis. The volcano plot shows the overall distribution of the differentially expressed lncRNAs (Fig. 5A). The heat map revealed that the differential expression of lncRNAs and Lnc NEAT1 was noticeably upregulated in H_2_O_2_-HUVEC-Exos (Fig. 5B). EPCs co-cultured with H_2_O_2_-HUVEC-Exos for 24 h maintained the highest level of Lnc NEAT1 in EPCs relative to the control and HUVEC-Exos groups (Fig. 5C,* P* < 0.01).

### H_2_O_2_-HUVEC-Exos affected the expression of proteins in the Wnt/β-catenin signaling pathway

Western blotting was used to analyze the effect of H_2_O_2_-HUVEC-Exos on β-catenin, c-myc, and cyclin D1 proteins in the Wnt/β-catenin signaling pathway. As shown in Fig. 5D, H_2_O_2_-HUVEC-Exos significantly increased the expression of β-catenin, c-myc, and cyclin D1 in comparison with the control and HUVEC-Exos groups (*P* < 0.05).

### Exosomal Lnc NEAT1 promoted the proliferation and invasion of EPCs by activating the Wnt/β-catenin signaling pathway

**Fig. 6:**
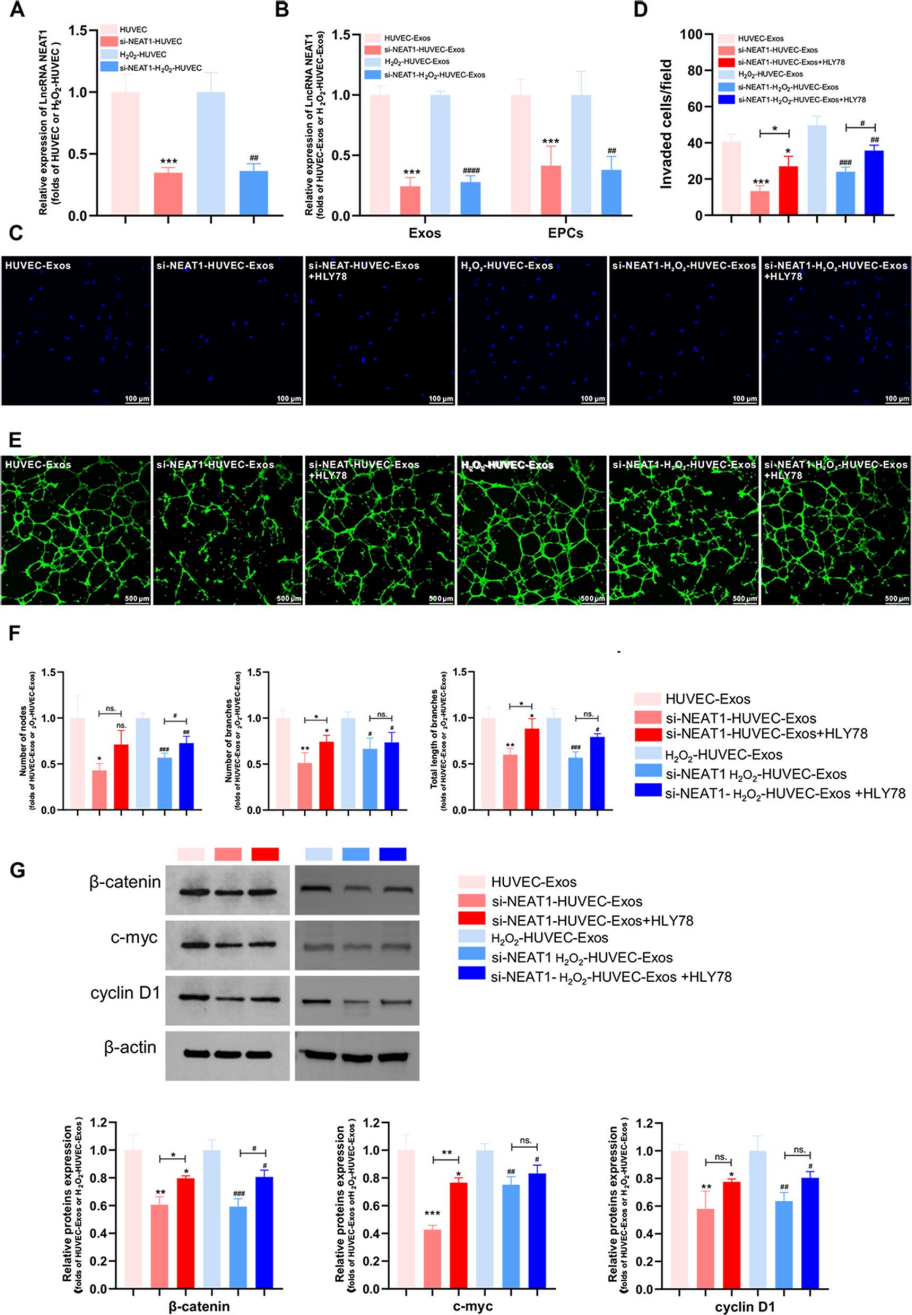
Exosomal Lnc NEAT1 accelerated angiogenesis via the Wnt/β-catenin pathway in EPCs. **A** The expression of Lnc NEAT1 in HUVECs and H_2_O_2_-HUVEC after transfection with si-NEAT1. *** *P* < 0.001 vs. HUVEC group, ^##^ *P* < 0.01 vs. H_2_O_2_-HUVEC-Exos group. **B** The expression of Lnc NEAT1 levels in Exos (HUVEC-Exos, si-NEAT1-HUVEC-Exos, H_2_O_2_-HUVEC-Exos and si-NEAT1-H_2_O_2_-HUVEC-Exos) and in EPCs after cultured with HUVEC-Exos, si-NEAT1-HUVEC-Exos, H_2_O_2_-HUVEC-Exos or si-NEAT1-H_2_O_2_-HUVEC-Exos. *** *P* < 0.001 vs. HUVEC-Exos group, ^##^ *P* < 0.01, ^####^ *P* < 0.0001 vs. H_2_O_2_-HUVEC-Exos group. **C** Representative images of invaded EPCs stained by DAPI after cultured with HUVEC-Exos, si-NEAT1-HUVEC-Exos, si-NEAT1-HUVEC-Exos + HLY78, H_2_O_2_-HUVEC-Exos, si-NEAT1- H_2_O_2_-HUVEC-Exos or si-NEAT1-H_2_O_2_-HUVEC-Exos + HLY78. **D** Quantitative analysis of (C). **E** Representative images of capillary-like tube formation generated by EPCs cultivated with HUVEC-Exos, si-NEAT1-HUVEC-Exos, si-NEAT1-HUVEC-Exos + HLY78, H_2_O_2_-HUVEC-Exos, si-NEAT1-H_2_O_2_-HUVEC-Exos, si-NEAT1-H_2_O_2_-HUVEC-Exos + HLY78 for 8 h. EPCs were labeled with Calcein-AM (green). **F** Quantitative analysis of (E). **G** The β-catenin, c-myc and cyclin D1 protein levels in EPCs after treated with HUVEC-Exos, si-NEAT1-HUVEC-Exos, si-NEAT1-HUVEC-Exos + HLY78, H_2_O_2_-HUVEC-Exos, si-NEAT1-H_2_O_2_-HUVEC-Exos, si-NEAT1-H_2_O_2_-HUVEC-Exos + HLY78C-Exos. ns. *P* > 0.05, * *P* < 0.05, ** *P* < 0.01, *** *P* < 0.001 versus HUVEC-Exos group, ns. *P* > 0.05, ^#^ *P* < 0.05, ^##^ *P* < 0.01, ^###^ *P* < 0.001 vs. H_2_O_2_-HUVEC-Exos group

To investigate the potential mechanisms of Lnc NEAT1, HUVECs and H_2_O_2_-HUVECs were transfected with Lnc NEAT1 siRNA. Knockdown efficiency was verified by RT-PCR (Fig. 6A). Compared with untreated HUVECs, the level of Lnc NEAT1 in exosomes derived from si-NEAT1 HUVECs (si-NEAT1-HUVEC-Exos) was markedly inhibited (Fig. 6B,* P *< 0.001). Simultaneously, the level of Lnc NEAT1 in si-NEAT1-H_2_O_2_-HUVEC-Exos was also significantly lower than that in H_2_O_2_-HUVEC-Exos (Fig. 6B, *P* < 0.0001). After EPCs were co-cultured with HUVEC-Exos, si-NEAT1-HUVEC-Exos, H_2_O_2_-HUVEC-Exos and si-NEAT1-H_2_O_2_-HUVEC-Exos for 24 h, RT-PCR was performed to determine the expression level of Lnc NEAT1 in EPCs, which was significantly decreased in the si-NEAT1-HUVEC-Exos and si-NEAT1-H_2_O_2_-HUVEC-Exos groups relative to the HUVEC-Exos and H_2_O_2_-HUVEC-Exos groups, respectively (Fig. 6B, *P* < 0.01 ).

To elucidate the effect of Lnc NEAT1 on angiogenesis of EPCs, transwell and tube formation assays were performed after EPCs were co-cultured with HUVEC-Exos, si-NEAT1-HUVEC-Exos, H_2_O_2_-HUVEC-Exos and si-NEAT1-H_2_O_2_-HUVEC-Exos. As expected, knockdown of Lnc NEAT1 both in HUVEC-Exos and H_2_O_2_-HUVEC-Exos significantly attenuated the invasion ability of EPCs, whereas this effect was partly reversed by co-treatment with HLY78 (an agonist of the Wnt/β-catenin signaling pathway) (Fig. 6C, D, *P* < 0.05). Moreover, when Lnc NEAT1 was blocked in HUVEC-Exos and H_2_O_2_-HUVEC-Exos, the tube formation capacity was significantly reduced (Fig. 6E, F, *P* < 0.05). HLY78 reversed the inhibitory effects of silencing of Lnc NEAT1 in HUVEC-Exos on the number of branches and total length of branches (*P* < 0.05) but not the number of nodes (Fig. 6E, F, *P* > 0.05). Conversely, compared to using si-NEAT1-H_2_O_2_-HUVEC-Exos alone, increased number of nodes (*P* < 0.05) but not the number of branches or total length of branches (Fig. 6E, F, *P* > 0.05) was observed in EPCs treated with si-NEAT1-H_2_O_2_-HUVEC-Exos + HLY78 combination. To explore the effects of Lnc NEAT1 on Wnt/β-catenin signaling pathway, the expression levels of β-catenin and its targets c-myc and cyclin D1 in EPCs were examined. Western blotting analysis indicated that exosomes from si-NEAT1-HUVECs and si-NEAT1-H_2_O_2_-HUVECs reduced the expression of the downstream genes of the Wnt/β-catenin pathway (Fig. 6G, *P* < 0.05). However, treatment with HLY78 partly revered this effect (Fig. 6G, *P* < 0.05).

### Lnc NEAT1 in H_2_O_2_-HUVEC-Exos enhanced the survival of skin flaps

**Fig. 7:**
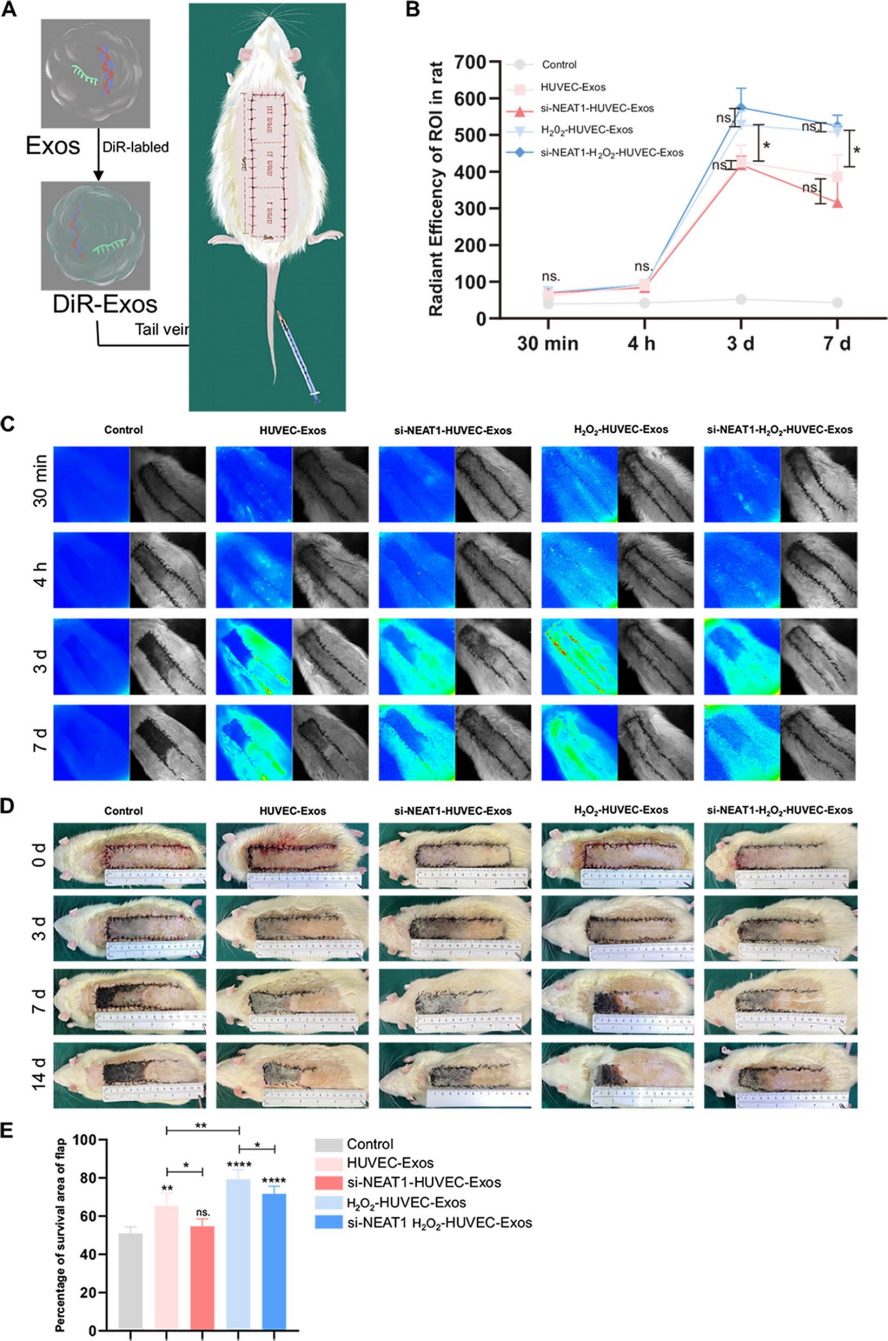
H_2_O_2_-HUVEC-Exos enhanced the survival of skin flaps. **A** Schematic representation of the experimental procedures. **B** Quantitative analysis of fluorescent signals intensity in control, HUVEC-Exos, si-NEAT1-HUVEC-Exos, H_2_O_2_-HUVEC-Exos and si-NEAT1-H_2_O_2_-HUVEC-Exos groups at 30 min, 4 h, 3 d and 7 d, *n* = 5 in each group. ns. *P* > 0.05, * *P* < 0.05. **C** The representative in vivo fluorescence imaging in different groups at 30 min, 4 h, 3 d and 7 d after the injection of HUVEC-Exos, si-NEAT1-HUVEC-Exos, H_2_O_2_-HUVEC-Exos, si-NEAT1-H_2_O_2_-HUVEC-Exos or PBS. **D** Representative gross appearance of viable and necrotic skin regions in skin flap treated in control, HUVEC-Exos, si-NEAT1-HUVEC-Exos, H_2_O_2_-HUVEC-Exos, si-NEAT1-H_2_O_2_-HUVEC-Exos groups. **E** Quantification of percentages of survival area on day 14. * *P* < 0.05, ** *P* < 0.01, **** *P* < 0.0001 vs. control group

To better understand the effects of exosomal Lnc NEAT1 in skin flap model rats, the distribution of DiR-labeled exosomes in rats was monitored by in vivo fluorescence imaging at 30 min, 4 h, 3 d, and 7 d after exosome injection. Few fluorescent signals were detected at 30 min and 4 h time points in all groups (Fig. 7B, C). The signals actively moved towards the skin flap, reached a peak on day 3, and then gradually decreased over time in the four exosome-treated groups. Additionally, the fluorescence signals of the H_2_O_2_-HUVEC-Exos and si-NEAT1-H_2_O_2_-HUVEC-Exos groups showed statistically higher intensity compared to the other three groups at 3 d and 7 d time points (Fig. 7A, B, *P* < 0.05). However, downregulation of Lnc NEAT1 in HUVEC-Exos and H_2_O_2_-HUVEC-Exos exhibited no significant difference on the fluorescence signal around the flaps (Fig. 7A, B, *P* > 0.05). In contrast, no obvious fluorescent signal was detected in the control group at any of the observation time points.

Three days after surgery, the flaps of each group showed different degrees of skin swelling; the distal end was dark brown, but no obvious necrosis was observed. From day 7 to day 14 after surgery, areas II and III of the flap in the control, HUVEC-Exos groups, si-NEAT1-HUVEC-Exos and si-NEAT1-H_2_O_2_-HUVEC-Exos group had black crush, with poor elasticity and hard texture, while H_2_O_2_-HUVEC-Exos had pink sink in area II, with good elasticity, and black crust only in area III. In comparison, the proximal ends of the skin flaps in all five groups survived with normal color and texture. (Fig. 7D). The distal necrotic parts of the flaps were scabby and the hard texture with no hair growth. The degree of flap necrosis was the most serious in the control group. The boundary between the surviving and necrotic tissues is clear. Percentages of the survival area were evaluated 14 days after the operation. The percentage of flap survival area in the H_2_O_2_-HUVEC-Exos group was 79.3%, which was remarkably higher than that in the control group (51%), the HUVEC-Exos group (65.3%), the si-NEAT1-HUVEC-Exos group (62.33%) and si-NEAT1-H_2_O_2_-HUVEC-Exos group (77.33%) (Fig. 7E, *P* < 0.05). Furthermore, when Lnc NEAT1 was knocked down in HUVEC-Exos and H_2_O_2_-HUVEC-Exos, the survival area of flaps was significantly decreased compared to untreated HUVEC-Exos and H_2_O_2_-HUVEC-Exos, respectively (Fig. 7D, *P* < 0.05).

#### H_2_O_2_-HUVEC-Exos increased neovascularization of skin flaps and homing of EPCs

**Fig. 8:**
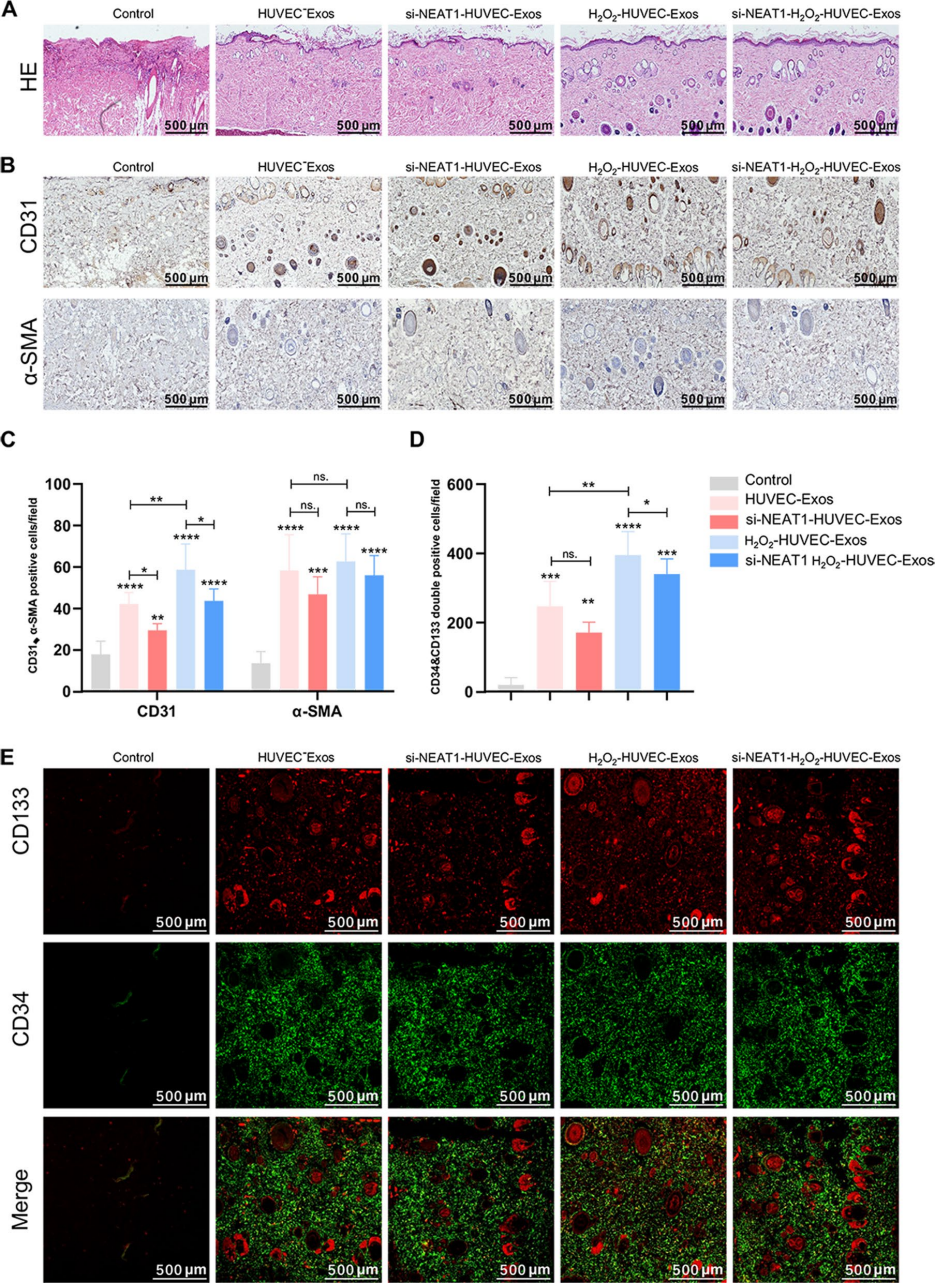
H_2_O_2_-HUVEC-Exos increased neovascularization of flaps and recruitment of EPCs. **A** H&E staining of three groups of skin flaps. **B** The representative immunohistochemistry staining of CD31 and α-SMA in control, HUVEC-Exos, si-NEAT1-HUVEC-Exos, H_2_O_2_-HUVEC-Exos, si-NEAT1-H_2_O_2_-HUVEC-Exos groups. **C.** Quantitative analysis of CD31 and α-SMA positive vessel number. **D** Quantitative analysis of the number of CD34/CD133 double positive cells. **E** The representative immunofluroscence staining of CD133 and CD34 positive cells. CD133 positive cells were shown in red. CD34 positive cells were shown in green. ns. *P* > 0.05, * *P* < 0.05, ** *P* < 0.01, *** *P* < 0.001, **** *P* < 0.0001 vs. control group

Fourteen days after surgery, H&E staining and immunohistochemistry were performed. As shown in Fig. 8A, the control group displayed many infiltrated inflammatory cells and few microvessels. In contrast, the H_2_O_2_-HUVEC-Exos and si-NEAT1-H_2_O_2_-HUVEC-Exos groups exhibited complete epithelialization with a hierarchical structure, a large number of blood vessels, and few diffused neutrophil infiltration compared to the other groups. Newly formed vessels and mature vessels were characterized by CD31 and α-SMA staining (Fig. 8B), respectively. Compared with the control group, the number of CD31 and α-SMA positive vessels increased significantly in the other four groups treated with exosomes (Fig. 8B, C,* P* < 0.01). However, the number of CD31 positives vessels in the H_2_O_2_-HUVEC-Exos group was higher than that of HUVEC-Exos group (Fig. 8B, C, *P* < 0.01). Moreover, less number of CD31-positive vessels was observed after Lnc NEAT1 was silenced in both HUVEC-Exos and H_2_O_2_-HUVEC-Exos (Fig. 8B, C, *P* < 0.05). Interestingly, there was no significant difference in the number of α-SMA positive vessels between the HUVEC-Exos and the H_2_O_2_-HUVEC-Exos groups (*P* > 0.05). Similarly, exosomes from si-NEAT1-HUVECs and si-NEAT1-H_2_O_2_-HUVECs did not significantly reduce the number of α-SMA-positive blood vessels compared with exosomes from untreated HUVECs and H_2_O_2_-HUVECs, respectively (Fig. 8B, C, *P* > 0.05). The number of CD34/CD133 double-positive cells, which are markers of EPCs, was noticeably higher in the H_2_O_2_-HUVEC-Exos group than that in the other groups (Fig. 8D, E, *P* < 0.05). Furthermore, inhibition of Lnc NEAT1 in H_2_O_2_-HUVEC-Exos resulted in a dramatic reduction in the homing number of EPCs (*P* < 0.05), but there was no significant difference in the number of EPCs homing between HUVEC-Exos and si-NEAT1-HUVEC-Exos groups (Fig. 8D, E, *P* > 0.05).

## Discussion

Recently, exosomes have garnered widespread attention as emerging therapies against ischemic diseases [27]. Currently, flap necrosis remains a challenge because of inflammation, blood supply deficiency, and oxidative stress [28]. To date, few studies have focused on the effect of exosomes derived from HUVECs under oxidative stress on flap survival. This study demonstrated for the first time that exosomes derived from HUVECs stimulated by oxidative stress significantly promote the pro-angiogenic ability of EPCs through the Wnt/β-catenin signaling pathway mediated by Lnc NEAT1 in vitro and enhanced random flap survival by promoting angiogenesis in vivo.

Ischemia is a common complication after flap surgery [29]. With the restoration of blood supply to the flap, the tissue may undergo ischemia–reperfusion injury [30, 31]. As a double-edged sword, excessive production of reactive oxygen species (ROS) leads to inflammatory infiltration of neutrophils, increased secretion of proteases, and a large amount of oxidative intermediate products, which ultimately causes cell death [32]. However, low levels of ROS as a signal molecule directly regulate the activity of transcription factors, leading to a reduction in apoptosis [33, 34]. Previous reports have shown that low-dose H_2_O_2_ can induce cells to tolerate a higher degree of oxidation, which could have a protective effect against harmful conditions, and prevent the induction of apoptosis [34, 35]. Exposure to the oxidative stress microenvironment not only increases the expression of pro-angiogenic proteins in stimulated cells, but also increases the expression of related genes in exosomes [36–38]. Accordingly, we used a low-concentration H_2_O_2_ to effect HUVECs for 12 h to establish an appropriate oxidative stress model to mimic the in vivo ischemic microenvironment.

It has been suggested that the underlying mechanisms of cell transplantation therapy are likely dependent on the paracrine activity of cells [39]. Recent studies have shown that cells transfer exosomes to stem cells via paracrine mechanisms, thereby regulating their functions [40]. It is believed that treatment with exosomes may overcome the limitations and risks associated with cell transplantation therapy [41]. Liu et al. demonstrated that microvesicles from endothelial cells under oxidative stress promoted angiogenesis by transferring miR-92a-3p to recipient endothelial cells [38]. In addition, exosomes secreted by retinal pigment epithelium exposed to oxidative stress not only significantly increased the levels of VEGF protein and certain mRNAs, but also remarkably enhanced the pro-angiogenic function of endothelial cells [36]. Similarly, our study confirmed that the exosomes derived from HUVECs stimulated by H_2_O_2_ have a strong capacity to promote tube formation, migration, and proliferation of EPCs in a dose-dependent manner. EPCs in the circulation are known to participate in the formation and repair of blood vessels by recruiting to the ischemic site [42, 43]. Damaged vascular endothelial cells may activate platelets and vascular smooth muscle cells and secrete chemokines to recruit EPCs from the circulation [44, 45]. Meanwhile, through the chemokine receptors in the exfoliated endothelial area, EPCs can adhere to the injured site to take part in angiogenesis and inhibit intimal hyperplasia [46, 47]. In addition, we have provided new evidence that H_2_O_2_-HUVEC-Exos significantly increased mRNA levels of pro-angiogenic genes (VEGPA and PDGFC) and decreased the expression of anti-angiogenic genes (PTEN) in EPCs. Notably, there were no significant differences between H_2_O_2_-HUVEC-Exos and HUVEC-Exos with regard to surface marker expression, size, and shape. These phenomena indicate that the superior proangenic ability of exosomes conferred by preconditioning with H_2_O_2_ may be mainly attributed to the improvement in quality and activity of substances in their cargo, further promoting tube formation, migration, and proliferation of EPCs.

Recent evidence indicates that endothelia cell-Exos contain multiple bioactive molecules, which participate in the regulation of cell, growth, migration, and vascular development [48]. Since EPCs internalized large amounts of HUVEC exosomes (Fig. 3D), suggesting exosomes could be a potential shuttle for delivering bioactive contents from HUVECs to EPCs, thereby changing their behavior. Among the major contents in exosomes, lncRNAs attract particular attention and play an important role in a variety of biological processes [49, 50]. Here, we compared the lncRNA profiles in HUVEC exosomes and found that Lnc NEAT1 expression was remarkably elevated in the H_2_O_2_-HUVEC-Exos, indicating that LncNEAT1 was endogenous cargo of HUVEC-derived exosomes, which was consistent with the previous evidence that high expression of Lnc NEAT1 could be induced under hypoxic conditions, leading to a positive feedback loop that promotes cell proliferation, colony formation, and reduced cell apoptosis [51, 52]. Lnc NEAT1 is an essential component of nuclear paraspeckles and mainly functions as a transcriptional regulator. It has been reported that Lnc NEAT1 regulated the occurrence of hemangiomas and promoted breast cancer cell survival [53]. In addition, high levels of Lnc NEAT1 are associated with malignant progression in glioma and poor clinical prognosis [54]. However, other studies have shown that Lnc NEAT1 acts as a tumor suppressor [55]. Therefore, the role of Lnc NEAT1 may be cell-type dependent, which has not been fully elucidated. Next, we sought to identify the underlying molecular mechanisms by which Lnc NEAT1 regulates downstream effectors. In our study, we found that silence of Lnc NEAT1 in both HUVEC-Exos and H_2_O_2_-HUVEC-Exos significantly suppressed EPCs migration and tube formation. To explore whether Lnc NEAT1 affects EPCs angiogenesis through Wnt/β-catenin signaling pathway, western blot analysis was used to detect the protein level associated with Wnt/β-catenin pathway after Lnc NEAT1 knockdown. The result revealed that downregulated Lnc NEAT1 in both HUVEC-Exos and H_2_O_2_-HUVEC-Exos repressed the Wnt/β-catenin signaling pathway, leading to downregulation of the protein levels of β-catenin, c-myc, and cyclin D1. With the use of a Wnt activator, the inhibitory effect of Lnc NEAT1 on EPCs function was partially alleviated. The phenomenon could be deciphered that Lnc NEAT1 participate in angiogenesis through multiple signaling pathways [56–58]. Yangwei Xu et al.[56] demonstrated that LncRNA NEAT1 promoted gastric cancer progression through miR-17-5p/TGFβR2 axis up-regulated angiogenesis. Lidong Zhao et al.[57] also found that Lnc NEAT1 promoted trophoblast proliferation and invasion in gestational hypertension and alleviated vascular endothelial injury by inhibiting miRNA-205-5p. Therefore, Wnt activators could only activate the Wnt/β-catenin signaling pathway, rendering them incapable of fully reversing the inhibition of EPC function caused by Lnc NEAT1 silencing. The Wnt/β-catenin signaling pathway is associated with various processes involved in the regulation of stem cell self-renewal, differentiation, and cell reprogramming [59]. In accordance with our study, Lnc NEAT1 activates Wnt/β-catenin signaling and contributes to colorectal cancer progression via DDX5 [60]. Moreover, c-myc and cyclin D1 are direct targets of the Wnt/β-catenin signaling pathway and can regulate angiogenesis by increasing the expression of VEGF [61, 62]. C-myc promotes cell proliferation and immortalization [63]. Besides, c-myc ablation impairs endothelial cell proliferation and mitochondrial function, but its specific overexpression in endothelial cells promotes these processes [64]. As a member of the cyclin family, cyclin D1 is capable of regulating EPC proliferation [65]. To the best of our knowledge, there are few studies on Lnc NEAT1 regulating the function of EPC. We are the first to confirm that Lnc NEAT1 can enhance the pro-angiogenic ability of EPCs by activating Wnt/β-catenin pathway and may act on downstream genes involved in angiogenesis.

To better understand the proangiogenic potential of the engineered exosomes, DiR-labeled four kinds of exosomes (HUVEC-Exos, si-NEAT1-HUVEC-Exos, H_2_O_2_-HUVEC-Exos and si-NEAT1-H_2_O_2_-HUVEC-Exos) were used to evaluate their effects on skin flap survival in a rat model. Interestingly, the fluorescence signal around the flap gradually increased over time and reached a peak on the third day. Exosomes derived from HUVECs showed natural targeting ability, which could be further used as nanocarriers for delivering different therapeutic molecules to reconstructive tissues. The phenomenon of exosomes gathering at the injury site is analogous to stem cell homing through adhesion factors and receptors on their surface [66, 67]. Coincidentally, mesenchymal stem cell-derived exosomes could recruit around the wound and promote wound healing after injection into mice through the tail vein and specifically accumulate in the acute kidney injury model [66]. Additionally, after 3 and 7 days of exosomes injection into rats, the fluorescence signal around the skin flap in H_2_O_2_-HUVEC-Exos and si-NEAT1-H_2_O_2_-HUVEC-Exos significantly increased. However, silencing Lnc NEAT1 in both HUVEC-Exos and H_2_O_2_-HUVEC-Exos did not affect the fluorescence signal intensity around the skin flap relative to that in HUVEC-Exos and H_2_O_2_-HUVEC-Exos, respectively. Previous study has showed that oxidative stress could activate the adhesion of RBCs to laminin by inducing post-translational modification of Lu/BCAM, which altered its distribution on the cell surface, resulting in aggregates with high binding potential to laminin [68]. Oxidative stress is one of the important pathogenesis of pancreatitis, leading to upregulation of adhesion molecules, which in turn accelerates disease progression [69]. H_2_O_2_-induced-oxidative stress may alter the number and distribution of adhesion molecules on the surface of exosomes, making them more likely to adhere to damaged sites and be taken up by EPCs. Gross observation of flap survival showed that all treated flaps achieved a remarkable decrease in flap necrosis and increased flap survival compared to that in the control, especially in H_2_O_2_-HUVEC-Exos. Consistent with the gross observation, H&E staining revealed that H_2_O_2_-HUVEC-Exos exhibited more complete epithelialization with a hierarchical structure and few diffused neutrophil infiltration compared to the other groups. Flaps have a unique process of neovascularization and vascular modeling for growth, maintenance, and regeneration [31]. Angiogenesis is known to play a critical role in flap survival and is required for tissue formation [39]. Rapid neovascularization to restore blood perfusion is very important to avoid skin flap necrosis [4, 5]. Our results showed that the use of exosomes, especially H_2_O_2_-HUVEC-Exos, greatly increased the generation of newly formed vessels, which corresponded to their strongest in vitro angiogenic ability. However, H_2_O_2_-HUVEC-Exos did not accelerate vessels maturation in the skin flap, possibly due to the limited observation period. It was clearly observed that CD133/CD34 double-positive cells, which were believed to be EPCs, diffusely infiltrated the flaps, especially in the H_2_O_2_-HUVEC-Exos group. Similarly, previous studies have shown that the application of hiPSC-MSC-Exos is potentially useful in treating certain ischemic diseases [39]. These encouraging results suggest that these special exosomes were recruited to the injured area after entering the blood circulation, and may target EPCs to promote angiogenesis, which gives superior results over the other groups.

## Conclusion

**Fig. 9:**
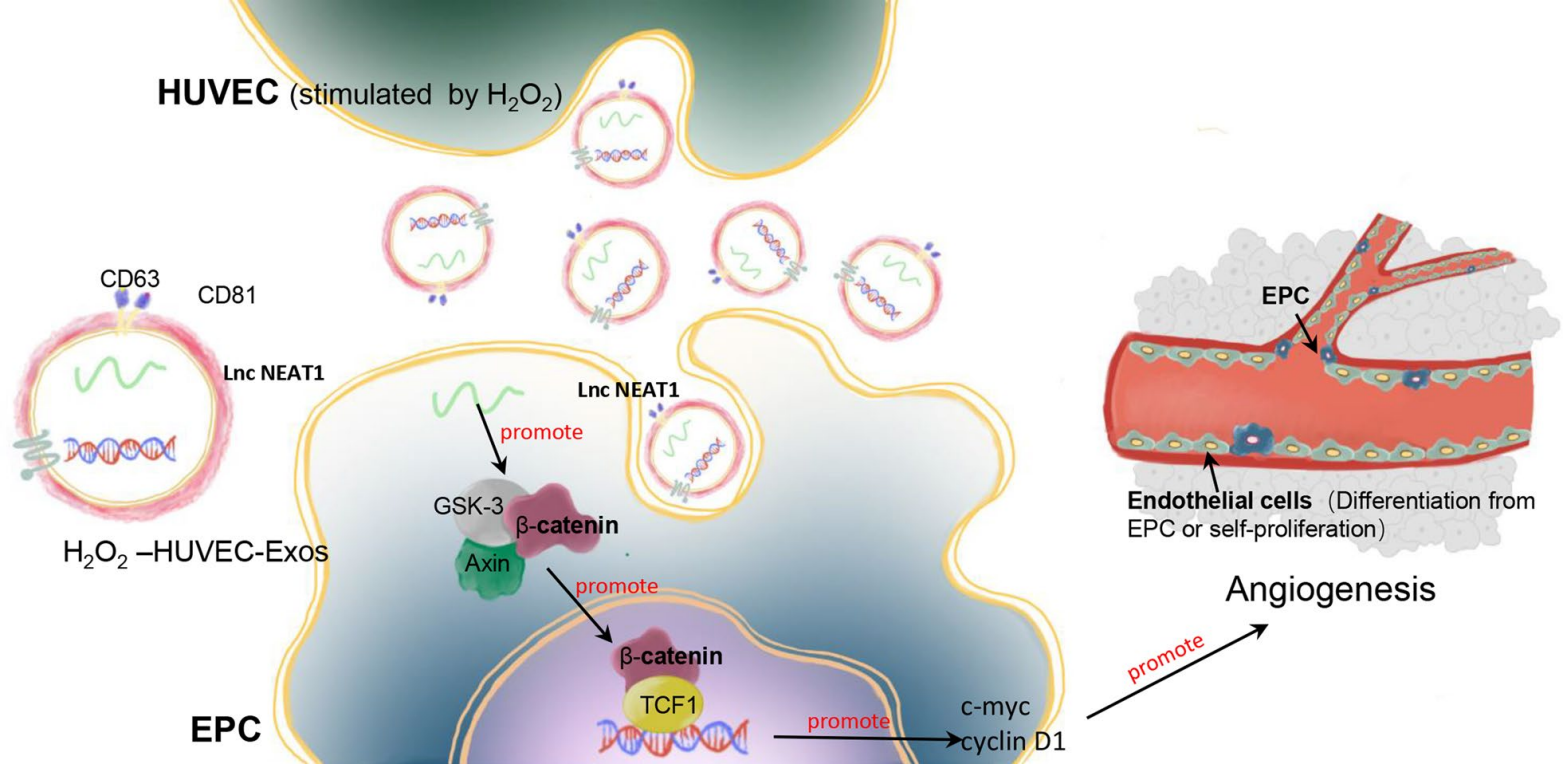
Schematic illustration of the function and mechanisms of HUVEC-Exos on EPCs

The present study shows that exosomes derived from HUVECs stimulated with an appropriate concentration of H_2_O_2_ promoted neovascularization and increased the survival rate of skin flaps by enhancing the angiogenic ability of EPCs. Furthermore, we demonstrated the important role of exosomal Lnc NEAT1 in EPC function via the Wnt/β-catenin pathway, which might influence the underlying molecular mechanisms that promote angiogenesis (Fig. 9). These findings highlight that the oxidative stress-induced HUVEC exosomal Lnc NEAT1/Wnt/β-catenin axis in EPCs conferred a pro-angiogenic function in skin flap survival, which could be used as a novel therapeutic approach.

## Supplementary Information

**Additional file 1** Morphology, count and viability of HUVECs treated with or without serum and H_2_O_2_. A. Representative images of cell morphology in three groups at different time points. B. The proliferation HUVECs in three groups at different time points measured by CCK8 assay. C. Quantitative analysis of cell number in three groups at different time points.

**Additional file 2** The ratio of number and protein amount from HUVEC-Exos and H_2_O_2_-HUVEC-Exos.

**Additional file 3** Number and protein amount of HUVEC-Exos and H_2_O_2_-HUVEC-Exos.

## Data Availability

The datasets used and/or analyzed during the current study are available from the corresponding author on reasonable request.

## Abbreviations

HUVECs: Human vascular endothelial cells
EPCs: Endothelial progenitor cells
CCK8: Cell counting kit-8
qRT-PCR: Quantitative real-time polymerase chain reaction
lncRNAs: Long non-coding RNAs
Lnc NEAT1: LncRNA enrichment enriched transcript
VEGF: Vascular endothelial growth factors
miRNA: MicroRNAs
ncRNAs: Non-coding RNAs
SOD: Superoxide dismutase
FITC: Fluorescein isothiocyanate
PE: Phycoerythrin
APC: Allophycocyanin
DAPI: 4',6-Diamidino-2-phenylindole
RIPA: Radio-immunoprecipitation assay
NTA: Nanoparticle tracking analysis
TEM: Transmission electron microscope
BCA: Bicinchoninic acid
PFA: Paraformaldehyde
PTA: Phosphotungstic acid
SDS-PAGE: Sodium dodecyl sulfate polyacrylamide gel electrophoresis
HRP: Horseradish peroxidase
GAPDH: Glyceraldehyde-3-phosphate dehydrogenase
VEGFA: Vascular endothelial growth factor A
PDGFC: Platelet derived growth factor C
PTEN: Prime time entertainment network
H&E: Hematoxylin and eosin
DAB: 3,3’-Diaminobenzidine tetrahydrochloride

## References

[1] Lee MS, Ahmad T, Lee J (2017). Dual delivery of growth factors with coacervate-coated poly(lactic-co-glycolic acid) nanofiber improves neovascularization in a mouse skin flap model. Biomaterials.

[2] Mayo JS, Kurata WE, O'Connor KM (2019). Oxidative stress alters angiogenic and antimicrobial content of extracellular vesicles and improves flap survival. Plast Reconstr Surg Glob Open.

[3] Chen T, Tu Q, Cheng L (2018). Effects of curculigoside a on random skin flap survival in rats. Eur J Pharmacol.

[4] Martin P (1997). Wound healing–aiming for perfect skin regeneration. Science.

[5] Griffith LG, Naughton G (2002). Tissue engineering–current challenges and expanding opportunities. Science.

[6] Pedretti S, Rena CL, Orellano L (2020). Benefits of pentoxifylline for skin flap tissue repair in rats. Acta Cir Bras.

[7] Street J, Bao M, DeGuzman L (2002). Vascular endothelial growth factor stimulates bone repair by promoting angiogenesis and bone turnover. Proc Natl Acad Sci U S A.

[8] Lin J, Jia C, Wang Y (2019). Therapeutic potential of pravastatin for random skin flaps necrosis: involvement of promoting angiogenesis and inhibiting apoptosis and oxidative stress. Drug Des Devel Ther.

[9] Lin R, Lin J, Li S (2018). Effects of the traditional Chinese medicine baicalein on the viability of random pattern skin flaps in rats. Drug Des Devel Ther.

[10] Fonseca P, Vardaki I, Occhionero A (2016). Metabolic and signaling functions of cancer cell-derived extracellular vesicles. Int Rev Cell Mol Biol.

[11] Sato-Kuwabara Y, Melo SA, Soares FA (2015). The fusion of two worlds: non-coding RNAs and extracellular vesicles–diagnostic and therapeutic implications (Review). Int J Oncol.

[12] Boelens MC, Wu TJ, Nabet BY (2014). Exosome transfer from stromal to breast cancer cells regulates therapy resistance pathways. Cell.

[13] Li Y, Zhang X, Zheng Q (2020). YAP1 inhibition in HUVECs is associated with released exosomes and increased hepatocarcinoma invasion and metastasis. Mol Ther Nucleic Acids.

[14] Zhang Y, Qin Y, Chopp M (2020). Ischemic cerebral endothelial cell-derived exosomes promote axonal growth. Stroke.

[15] Li J, Tian H, Yang J (2016). Long noncoding RNAS regulate cell growth, proliferation, and apoptosis. DNA Cell Biol.

[16] Ali T, Grote P (2020). Beyond the RNA-dependent function of LncRNA genes. eLife.

[17] Dong P, Xiong Y, Yue J (2018). Long non-coding RNA NEAT1: a novel target for diagnosis and therapy in human tumors. Front Genet.

[18] Fan JT, Zhou ZY, Luo YL (2021). Exosomal lncRNA NEAT1 from cancer-associated fibroblasts facilitates endometrial cancer progression via miR-26a/b-5p-mediated STAT3/YKL-40 signaling pathway. Neoplasia.

[19] Yan H, Wang Z, Sun Y (2021). Cytoplasmic NEAT1 suppresses aml stem cell self-renewal and leukemogenesis through inactivation of wnt signaling. Adv Sci.

[20] Cui Y, Yin Y, Xiao Z (2019). LncRNA Neat1 mediates miR-124-induced activation of Wnt/β-catenin signaling in spinal cord neural progenitor cells. Stem Cell Res Ther.

[21] Chen Y, Cao J, Peng W (2020). Neurotrophin-3 accelerates reendothelialization through inducing EPC mobilization and homing. Open Life Sci.

[22] Simard T, Jung RG, Motazedian P (2017). Progenitor cells for arterial repair: incremental advancements towards therapeutic reality. Stem Cells Int.

[23] Yoder MC (2012). Human endothelial progenitor cells. Cold Spring Harb Perspect Med.

[24] Reynolds JA, Robertson AC, Bruce IN (2014). Improving cardiovascular outcomes in rheumatic diseases: therapeutic potential of circulating endothelial progenitor cells. Pharmacol Ther.

[25] Yang Z, von Ballmoos MW, Faessler D (2010). Paracrine factors secreted by endothelial progenitor cells prevent oxidative stress-induced apoptosis of mature endothelial cells. Atherosclerosis.

[26] Li WD, Zhou DM, Sun LL (2018). LncRNA WTAPP1 promotes migration and angiogenesis of endothelial progenitor cells via MMP1 through microrna 3120 and Akt/PI3K/autophagy Pathways. Stem Cells.

[27] Yaghoubi S, Najminejad H, Dabaghian M (2020). How hypoxia regulate exosomes in ischemic diseases and cancer microenvironment?. IUBMB Life.

[28] Jiang R, Lin C, Jiang C (2020). Nobiletin enhances the survival of random pattern skin flaps: involvement of enhancing angiogenesis and inhibiting oxidative stress. Int Immunopharmacol.

[29] Fukunaga Y, Izawa-Ishizawa Y, Horinouchi Y (2017). Topical application of nitrosonifedipine, a novel radical scavenger, ameliorates ischemic skin flap necrosis in a mouse model. Wound Repair Regen.

[30] Harder Y, Amon M, Laschke MW (2008). An old dream revitalised: preconditioning strategies to protect surgical flaps from critical ischaemia and ischaemia-reperfusion injury. J Plast Reconstr Aesthet Surg.

[31] Fang M, He J, Ma X (2020). Protective effects of dexmedetomidine on the survival of random flaps. Biomed Pharmacother.

[32] Huang YJ, Nan GX (2019). Oxidative stress-induced angiogenesis. J Clin Neurosci.

[33] Burton GJ, Jauniaux E (2011). Oxidative stress. Best Pract Res Clin Obstet Gynaecol.

[34] Reuter S, Gupta SC, Chaturvedi MM (2010). Oxidative stress, inflammation, and cancer: how are they linked?. Free Radic Biol Med.

[35] Zhang J, Chen GH, Wang YW (2012). Hydrogen peroxide preconditioning enhances the therapeutic efficacy of Wharton's Jelly mesenchymal stem cells after myocardial infarction. Chin Med J.

[36] Atienzar-Aroca S, Flores-Bellver M, Serrano-Heras G (2016). Oxidative stress in retinal pigment epithelium cells increases exosome secretion and promotes angiogenesis in endothelial cells. J Cell Mol Med.

[37] Chen J, Liu Z, Hong MM (2014). Proangiogenic compositions of microvesicles derived from human umbilical cord mesenchymal stem cells. PLoS ONE.

[38] Liu Y, Li Q, Hosen MR (2019). Atherosclerotic conditions promote the packaging of functional microrna-92a-3p into endothelial microvesicles. Circ Res.

[39] Zhang J, Guan J, Niu X (2015). Exosomes released from human induced pluripotent stem cells-derived MSCs facilitate cutaneous wound healing by promoting collagen synthesis and angiogenesis. J Transl Med.

[40] Han C, Sun X, Liu L (2016). Exosomes and their therapeutic potentials of stem cells. Stem Cells Int.

[41] Jung JH, Fu X, Yang PC (2017). Exosomes generated from ipsc-derivatives: new direction for stem cell therapy in human heart diseases. Circ Res.

[42] Hecht N, Schneider UC, Czabanka M (2014). Endothelial progenitor cells augment collateralization and hemodynamic rescue in a model of chronic cerebral ischemia. J Cereb Blood Flow Metab.

[43] Ishida Y, Kimura A, Kuninaka Y (2012). Pivotal role of the CCL5/CCR5 interaction for recruitment of endothelial progenitor cells in mouse wound healing. J Clin Invest.

[44] Zernecke A, Schober A, Bot I (2005). SDF-1alpha/CXCR4 axis is instrumental in neointimal hyperplasia and recruitment of smooth muscle progenitor cells. Circ Res.

[45] Hristov M, Zernecke A, Bidzhekov K (2007). Importance of CXC chemokine receptor 2 in the homing of human peripheral blood endothelial progenitor cells to sites of arterial injury. Circ Res.

[46] Wysocki SJ, Zheng MH, Smith A (1996). Monocyte chemoattractant protein-1 gene expression in injured pig artery coincides with early appearance of infiltrating monocyte/macrophages. J Cell Biochem.

[47] Fujiyama S, Amano K, Uehira K (2003). Bone marrow monocyte lineage cells adhere on injured endothelium in a monocyte chemoattractant protein-1-dependent manner and accelerate reendothelialization as endothelial progenitor cells. Circ Res.

[48] Todorova D, Simoncini S, Lacroix R (2017). Extracellular vesicles in angiogenesis. Circ Res.

[49] Ferrè F, Colantoni A, Helmer-Citterich M (2016). Revealing protein-lncRNA interaction. Brief Bioinform.

[50] Li X, Wu Z, Fu X (2014). lncRNAs: insights into their function and mechanics in underlying disorders. Mutat Res Rev Mutat Res.

[51] Choudhry H, Albukhari A, Morotti M (2015). Tumor hypoxia induces nuclear paraspeckle formation through HIF-2α dependent transcriptional activation of NEAT1 leading to cancer cell survival. Oncogene.

[52] Zheng X, Zhang Y, Liu Y (2018). HIF-2α activated lncRNA NEAT1 promotes hepatocellular carcinoma cell invasion and metastasis by affecting the epithelial-mesenchymal transition. J Cell Biochem.

[53] Yu L, Shu H, Xing L (2020). Silencing long non-coding RNA NEAT1 suppresses the tumorigenesis of infantile hemangioma by competitively binding miR-33a-5p to stimulate HIF1α/NF-κB pathway. Mol Med Rep.

[54] Zhou K, Zhang C, Yao H (2018). Knockdown of long non-coding RNA NEAT1 inhibits glioma cell migration and invasion via modulation of SOX2 targeted by miR-132. Mol Cancer.

[55] Idogawa M, Ohashi T, Sasaki Y (2017). Long non-coding RNA NEAT1 is a transcriptional target of p53 and modulates p53-induced transactivation and tumor-suppressor function. Int J Cancer.

[56] Xu Y, Li Y, Qiu Y (2021). LncRNA NEAT1 promotes gastric cancer progression through miR-17-5p/TGFβR2 Axis Up-regulated angiogenesis. Front Cell Dev Biol.

[57] Zhao L, Xiong M, Liu Y (2021). Baicalin enhances the proliferation and invasion of trophoblasts and suppresses vascular endothelial damage by modulating long non-coding RNA NEAT1/miRNA-205-5p in hypertensive disorder complicating pregnancy. J Obstet Gynaecol Res.

[58] Yuan J, Yi K, Yang L (2021). LncRNA NEAT1 promotes proliferation of ovarian cancer cells and angiogenesis of co-incubated human umbilical vein endothelial cells by regulating FGF9 through sponging miR-365: an experimental study. Medicine.

[59] Miki T, Yasuda SY, Kahn M (2011). Wnt/β-catenin signaling in embryonic stem cell self-renewal and somatic cell reprogramming. Stem Cell Rev Rep.

[60] Zhang M, Weng W, Zhang Q (2018). The lncRNA NEAT1 activates Wnt/β-catenin signaling and promotes colorectal cancer progression via interacting with DDX5. J Hematol Oncol.

[61] Ma S, Lu CC, Yang LY (2018). ANXA2 promotes esophageal cancer progression by activating MYC-HIF1A-VEGF axis. J Exp Clin Cancer Res.

[62] von Rahden BH, Stein HJ, Pühringer-Oppermann F (2006). c-myc amplification is frequent in esophageal adenocarcinoma and correlated with the upregulation of VEGF-A expression. Neoplasia.

[63] Huang X, Sun J, Chen G (2019). Resveratrol promotes diabetic wound healing via SIRT1-FOXO1-c-Myc signaling pathway-mediated angiogenesis. Front Pharmacol.

[64] Baudino TA, McKay C, Pendeville-Samain H (2002). c-Myc is essential for vasculogenesis and angiogenesis during development and tumor progression. Genes Dev.

[65] Qiu C, Xie Q, Zhang D (2014). GM-CSF induces cyclin D1 expression and proliferation of endothelial progenitor cells via PI3K and MAPK signaling. Cell Physiol Biochem.

[66] Grange C, Tapparo M, Bruno S (2014). Biodistribution of mesenchymal stem cell-derived extracellular vesicles in a model of acute kidney injury monitored by optical imaging. Int J Mol Med.

[67] Hu L, Wang J, Zhou X (2016). Exosomes derived from human adipose mensenchymal stem cells accelerates cutaneous wound healing via optimizing the characteristics of fibroblasts. Sci Rep.

[68] Lizarralde-Iragorri MA, Lefevre SD, Cochet S (2021). Oxidative stress activates red cell adhesion to laminin in sickle cell disease. Haematologica.

[69] Sato T, Shibata W, Maeda S (2019). Adhesion molecules and pancreatitis. J Gastroenterol.

