# Supplementary figures and images for: Oxidative stress-induced endothelial cells-derived exosomes accelerate skin flap survival through Lnc NEAT1-mediated promotion of endothelial progenitor cell function
Source: Stem Cell Res Ther. 2022 Jul 18;13:325. doi: 10.1186/s13287-022-03013-9 (PMC9290268; doi:10.1186/s13287-022-03013-9)

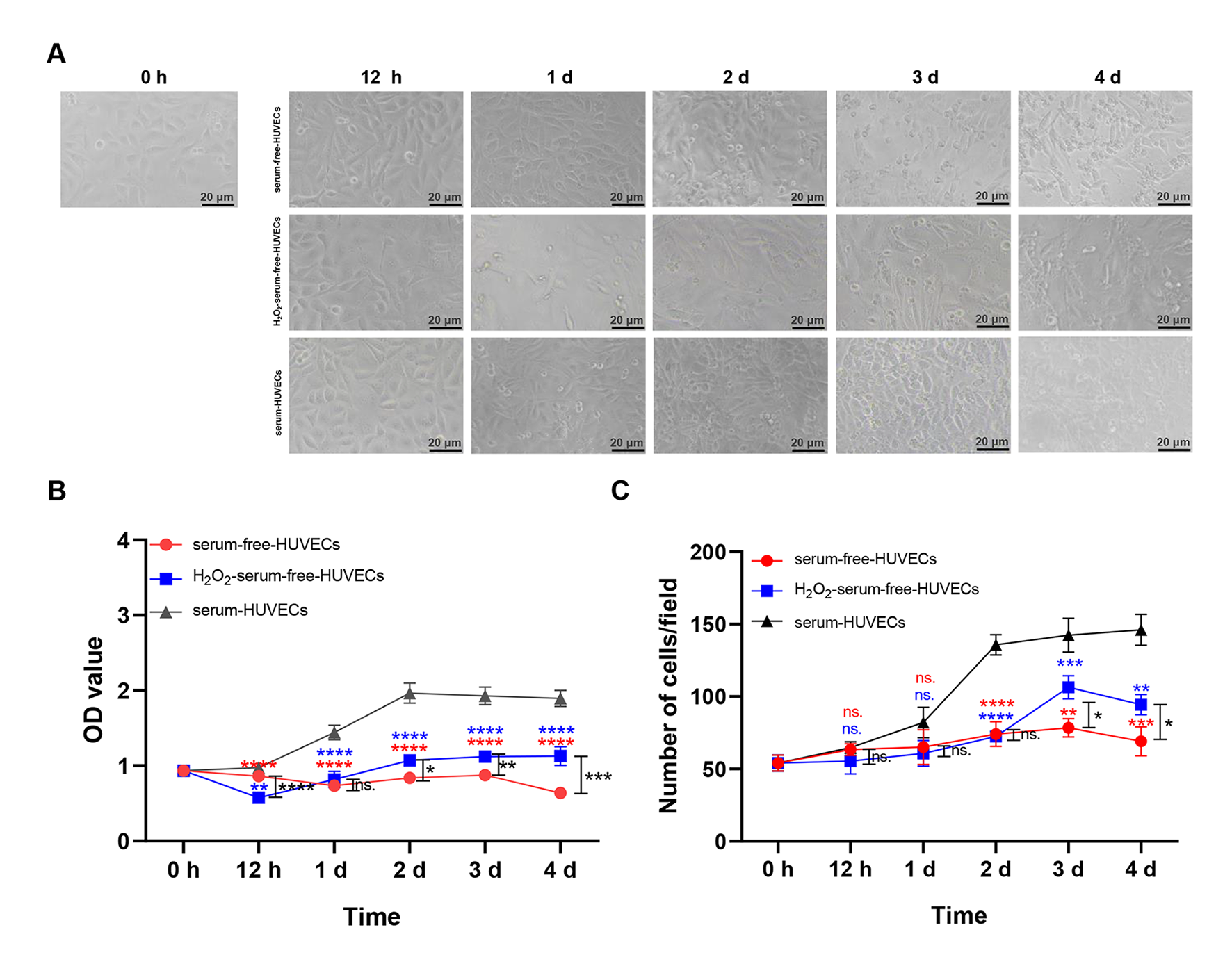

Supplement: Supplementary file 1 — Additional file 1 Morphology, count and viability of HUVECs treated with or without serum and H2O2. A. Representative images of cell morphology in three groups at different time points. B. The proliferation HUVECs in three groups at different time points measured by CCK8 assay. C. Quantitative analysis of cell number in three groups at different time points. [file 13287_2022_3013_MOESM1_ESM.tif]

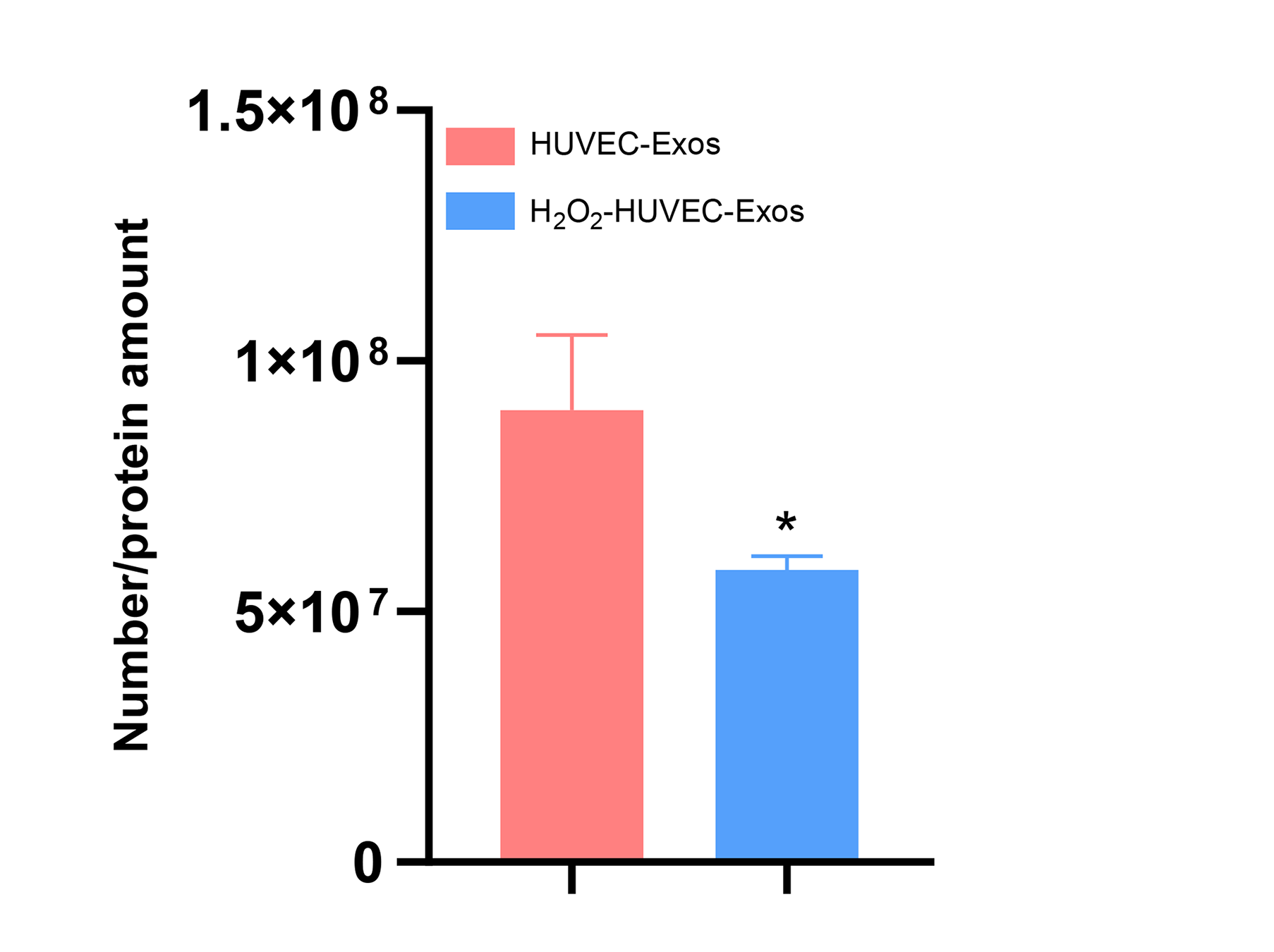

Supplement: Supplementary file 2 — Additional file 2 The ratio of number and protein amount from HUVEC-Exos and H2O2-HUVEC-Exos. [file 13287_2022_3013_MOESM2_ESM.tif]
