# Supplementary material for: Oxidative stress-induced endothelial cells-derived exosomes accelerate skin flap survival through Lnc NEAT1-mediated promotion of endothelial progenitor cell function
Source: Stem Cell Res Ther. 2022 Jul 18;13:325. doi: 10.1186/s13287-022-03013-9 (PMC9290268; doi:10.1186/s13287-022-03013-9)
Supplement: Supplementary file 3 — Additional file 3 Number and protein amount of HUVEC-Exos and H2O2-HUVEC-Exos. [file 13287_2022_3013_MOESM3_ESM.docx]

Supplemental Table 1: Number and protein amount of HUVEC-Exos and H_2_O_2_-HUVEC-Exos

| Sample | HUVEC-Exos | | | H_2_O_2_-HUVEC-Exos | | |
| --- | --- | --- | --- | --- | --- | --- |
|  | Number | Protein | ratio | Number | Protein(ug) | ratio |
| 1 | 4.0E+9 | 46.77 | 8.55E+7 | 4.6E+9 | 75.35 | 6.1E+7 |
| 2 | 4.2E+9 | 39.26 | 1.07E+8 | 4.7E+9 | 84.33 | 5.57E+7 |
| 3 | 3.9E+9 | 49.91 | 7.8E+7 | 4.4E+9 | 75.40 | 5.8E+7 |
